# Crystal structure of active CDK4-cyclin D and mechanistic basis for abemaciclib efficacy

**DOI:** 10.1038/s41523-022-00494-y

**Published:** 2022-11-29

**Authors:** Severine Isabelle Gharbi, Laura A. Pelletier, Alfonso Espada, Jesus Gutiérrez, Sonia Maria Gutiérrez Sanfeliciano, Charles T. Rauch, Maria Patricia Ganado, Carmen Baquero, Elisabet Zapatero, Aiping Zhang, Jordi Benach, Anna-Maria Russell, Leticia Cano, Sandra Gomez, Howard Broughton, Nicholas Pulliam, Carmen Maria Perez, Raquel Torres, Marjoke F. Debets, Alfonso de Dios, Oscar Puig, Mark T. Hilgers, Maria Jose Lallena

**Affiliations:** 1 Discovery Chemistry Research & Technology, Eli Lilly and Company, Madrid, Spain; 2 Lilly Biotechnology Center, Eli Lilly and Company, San Diego, CA USA; 3 Eli Lilly and Company, Indianapolis, IN USA; 4 Eli Lilly and Company, New York, NY USA

**Keywords:** Breast cancer, Tumour biomarkers

## Abstract

Despite the biological and therapeutic relevance of CDK4/6 for the treatment of HR+, HER2- advanced breast cancer, the detailed mode of action of CDK4/6 inhibitors is not completely understood. Of particular interest, phosphorylation of CDK4 at T172 (pT172) is critical for generating the active conformation, yet no such crystal structure has been reported to date. We describe here the x-ray structure of active CDK4-cyclin D3 bound to the CDK4/6 inhibitor abemaciclib and discuss the key aspects of the catalytically-competent complex. Furthermore, the effect of CDK4/6 inhibitors on CDK4 T172 phosphorylation has not been explored, despite its role as a potential biomarker of CDK4/6 inhibitor response. We show mechanistically that CDK4/6i stabilize primed (pT172) CDK4-cyclin D complex and selectively displace p21 in responsive tumor cells. Stabilization of active CDK4-cyclin D1 complex can lead to pathway reactivation following alternate dosing regimen. Consequently, sustained binding of abemaciclib to CDK4 leads to potent cell cycle inhibition in breast cancer cell lines and prevents rebound activation of downstream signaling. Overall, our study provides key insights demonstrating that prolonged treatment with CDK4/6 inhibitors and composition of the CDK4/6-cyclin D complex are both critical determinants of abemaciclib efficacy, with implications for this class of anticancer therapy.

## Introduction

Cyclin-dependent kinases 4 and 6 (CDK4/6) are integral for cell cycle regulation and progression through binding of D-type cyclins and phosphorylation of downstream proteins, including the tumor suppressor retinoblastoma (Rb) protein^1^. CDK-dependent Rb phosphorylation (pRb) results in increased expression of genes required for G1 progression^1^. Aberrant CDK4/6 activity and dysregulation of this signaling axis is frequent in multiple cancer types and it has emerged as an actionable drug target^1,2^. Several ATP-competitive CDK4/6 inhibitors are FDA approved for the treatment of advanced breast cancer (ABC)^3^. The CDK4/6 inhibitors, palbociclib and abemaciclib, in combination with aromatase inhibitors have demonstrated significantly improved progression-free survival in women with HR + , HER2- ABC^4,5^. These results were extended to include patients treated in combination with fulvestrant following endocrine therapy^6,7^, and for patients with node-positive early breast cancer at high risk of recurrence treatment with abemaciclib plus fulvestrant significantly improved invasive disease-free survival^8^.

Canonically, CDKs require cyclin binding and phosphorylation of the kinase activation segment (T-loop) for full catalytic activity^9,10^. However, the protein structures of CDK4 reported to date exhibit a conformation incompatible with catalysis and binding of ATP or ATP-competitive inhibitors^11–14^. While these reports were an important milestone in CDK4 structural biology (further discussed in Supplementary Table 1), the structure of active CDK4 has yet to be elucidated^13,14^ and our mechanistic understanding of CDK4/6 inhibitor-mediated sustained cell cycle inhibition is limited. Additionally, the effect of CDK4/6 inhibitors on CDK4 phosphorylation, a proposed biomarker of CDK4 activity and sensitivity to CDK4/6 inhibitors, has not been well-described^15^.

Activity of CDK4/6-cyclin D complexes are additionally regulated by the CDK cofactors CIP (p21)/KIP (p27 and p57) protein families^16^, which have been demonstrated to both inhibit and activate CDK4 dependent on biological conditions^17–19^. Furthermore, a recent study by Pack et al. reported using an engineered system that clinical CDK4/6 inhibitors (CDK4/6i) dissociated p21, but not p27, from trimeric cyclin D-CDK4-p21/p27 complexes resulting in rapid catalytic and non-catalytic inhibition of CDK4^20^. This was in contrast to a separate study which suggested that CDK4/6i required days of treatment to promote CDK4 inhibition^11,21^.

Here, we determine the first x-ray crystal structure of active CDK4 in complex with cyclin D3, in the presence of the CDK4/6 inhibitor abemaciclib. Additionally, we show in breast cancer cells that abemaciclib stabilizes primed CDK4-cyclin D complex and displaces p21, not observed in CDK6-cyclin D models, resulting in altered sensitivity in breast cancer models with greater CDK6 expression. The release of p21 from CDK4-cyclin D complex can then make it available to inhibit CDK2^20^. In addition, we show that continuous treatment with CDK4/6i is important to block rebound activation of CDK4 pathway. Overall, our results show that the composition of CDK4/6-cyclin D complex is critical in determining abemaciclib efficacy, treatment regimen and potential combination therapies.

## Results

### Crystal structure of active CDK4-cyclin D3 complex bound to abemaciclib

**Fig. 1:**
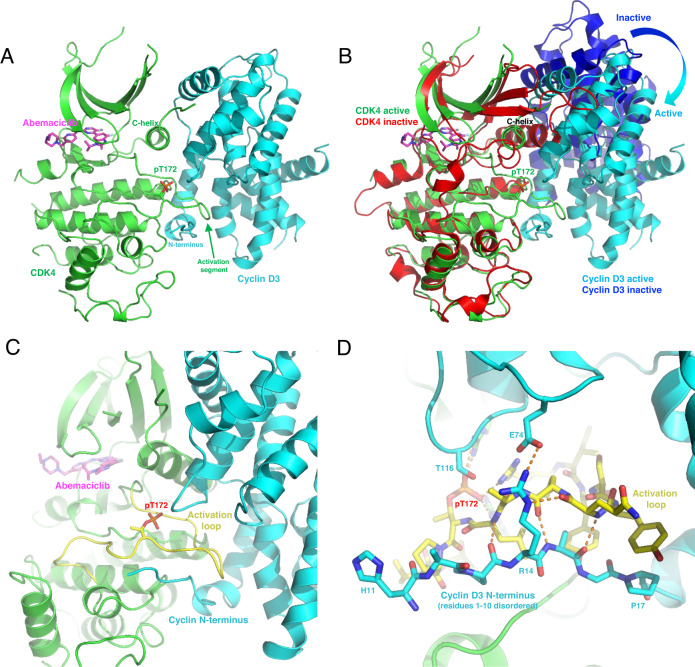
Crystal structure of active CDK4-cyclin D3 complex with abemaciclib bound. **A** The phosphorylated (pT172) complex exhibits the hallmarks of kinase activation, including an “out” activation segment, appropriately oriented C-helix, and an active-site competent for ligand binding (abemaciclib shown in magenta). **B** An example of an inactive complex (3G33.pdb), superposed with the active complex, with relative rotation indicated. Note the smaller CDK4-cyclin D3 interface and disordered cyclin D3 N-terminus. Extensive interaction of the activation segment (yellow) with the cyclin N-terminus. **C** The cyclin D3 N-terminus (ordered after residue H11) buttresses the activation segment, playing a role in adoption of the active conformation. **D** The interactions between kinase and cyclin involve an extensive Hydrogen bond network, largely composed of peptide backbone interactions. Residues R14 and E74 form an “electrostatic belt” around the activation segment.

In an effort to obtain crystals of the active CDK4-cyclin D complex, we chose to explore expression constructs that differed from those reported for previous structures^13^. For example, suspecting that the cyclin N-terminus might be important due to the presence of a pRb-binding motif we avoided affinity tags and other alterations in this region^22^. These efforts resulted in an abemaciclib-bound crystal structure of active CDK4-cyclin D3 complex of 2.5 Å (Supplementary Table 1 and 2). We determined the structure by molecular replacement with a model of active CDK6^23^ and cyclin D3^13^. The final refined model displayed the canonical features of an active CDK including an “in” αC-helix, ordered activation segment and unambiguous electron density at Threonine 172 that indicated phosphorylation (pT172), a state required for maximal CDK4 activity (Fig. 1A; Supplementary Figure 1)^16,24^.

Superposing the CDK4 kinase domain from this active complex to previously described conformationally inactive complexes demonstrated a rotation of cyclin D3 relative to active CDK4, and large conformational changes in CDK4 (Fig. 1A–D; Supplementary Figure 2; Supplementary Movie 1). This relative movement resulted in an increase in the buried surface between CDK4 and cyclin D, and a final structural arrangement similar to that observed in previously described CDK-cyclin active structures^16^. In contrast with the kinase domain, minimal structural changes to cyclin D3 were evident, except for the N-terminus, which becomes largely ordered (residues 11–22), and extensively interacts with the CDK4 activation segment (Fig. 1A–D). To verify the kinase activity of this active CDK4-cyclin D3 construct, we assessed the in vitro kinase activity of the recombinant complex, which demonstrated strong activity towards purified C-Terminal Rb Fragment (CTRF) (Supplementary Figure 3).

Many of the interactions shared between active CDK4 and abemaciclib, were analogous to those previously described in the monomeric abemaciclib-CDK6 structure (Supplementary Figure 4;^25^). These include direct hydrogen bonds between the aminopyrimidine ring and the hinge region of the kinase, the pyridyl and H96, and the free amine of the benzimidazole ring and K35.

### Abemaciclib binds CDK4 regardless of T172 phosphorylation

**Fig. 2:**
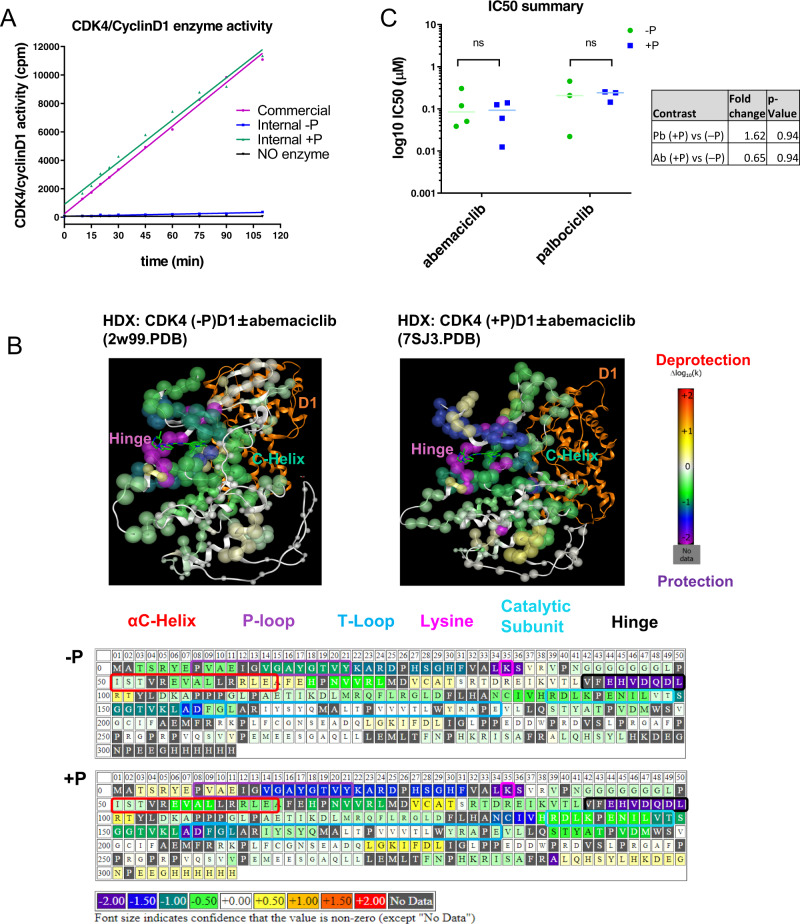
CDK4/6 inhibitor binds CDK4 regardless of T172 phosphorylation. **A** In vitro kinase activity assay: [γ-^33^P] ATP phosphorylation of CTRF using phosphorylated and unphosphorylated CDK4-cyclin D dimer. **B** (Top) HDX log10(k) projection (left panel (-P), 2W99.pdb) and phosphorylated (right panel (+P), 7SJ3.pdb) and (Bottom) differential HDX map of unphosphorylated (upper) and phosphorylated (lower) CDK4-cyclin D1 dimer and abemaciclib. The panels show the difference between the calculated log10 rate constants for the ligand-bound state and the Apo states (colors). Size of font/sphere provides an approximate indication of confidence that the value of the HDX perturbation is not zero; font color is chosen only for optimum contrast with the background. **C** in vitro target engagement assay with ATP/ADP desthiobiotin probes to measure abemaciclib (Ab) or palbociclib (Pb) affinity to unphosphorylated (-P) and phosphorylated (+P) CDK4-cyclin D recombinant proteins. ns – not significant. P-values determined by pairwise t-test. Error bars represent ± standard error.

Our crystal structure suggests that native pT172 is required for proper activation of CDK4 (Fig. 1). We measured CDK4 kinase activity with and without pT172 and as expected^13^, pT172 was required for CDK4 kinase activity (Fig. 2A). Next, to determine whether CDK4 T172 phosphorylation impacted the binding of abemaciclib for its target we used HDX-MS (Hydrogen Deuterium Exchange-Mass Spectrometry). Increasing protection in the “apo” states suggests less dynamic behavior of the protein in the HDX-protected region, while in the complex it indicates a strengthening of secondary structure in the bound conformation (Supplementary Figure 5 and 6). In the presence of abemaciclib, strong protection was evident in the hinge region of the phosphorylated and unphosphorylated state, suggesting abemaciclib bound the ATP pocket regardless of CDK4 T172 phosphorylation (Fig. 2B, Supplementary Figure 6).

To confirm the ability of abemaciclib to bind CDK4 regardless of T172 phosphorylation, we designed an in vitro assay using a modified ATP-probe to establish CDK4 affinity for ligand based on competition of the inhibitor with ATP. We found that both abemaciclib and palbociclib had affinity towards CDK4 irrespective of T172 phosphorylation (Fig. 2C). These observations demonstrate, in addition to inhibiting CDK4 activity, that abemaciclib binds CDK4 regardless T172 phosphorylation.

### Abemaciclib stabilizes primed CDK4 pT172 and prolonged treatment with abemaciclib is required for sustained inhibition of CDK4 signaling

**Fig. 3:**
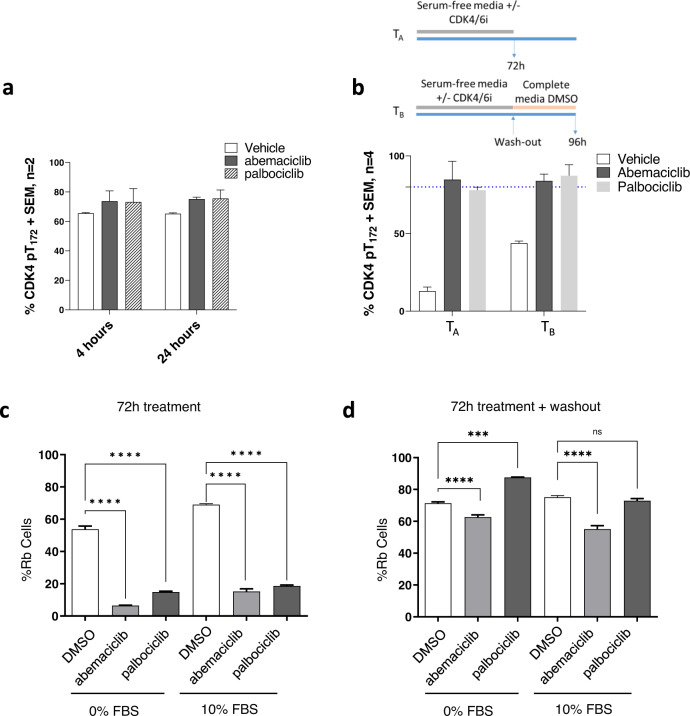
CDK4-T172 phosphorylation and CDK4/6 signaling in response to CDK4/6 inhibition. T47D cells were treated with vehicle or 1 μM CDK4/6 inhibitor for **a** 4 and 24 h in complete media (*n* = 2) or **b** following mitogen deprivation and wash out (*n* = 4). Cyclin D1 complexes were enriched and analyzed by LC-MS/MS. Quantification of CDK4-T172 phosphorylation is shown. Dashed blue line is baseline relative to (**a**). In (**b**) cell treatment is as described in the schematic. **c** Cells were treated in complete or serum-free media for 72 h with the indicated CDK4/6i (1 μM) and imaged for pRb (S780) content. **d** Cells were treated as in **c** followed by a 24 hour washout in complete media lacking inhibitors prior to pRb imaging. Rb protein phosphorylation was measured by high content imaging. **p < 0.01, ****p < 0.0001, ns – not significant. P-values determined by pairwise t-test. Error bars represent ± standard error.

To examine whether abemaciclib altered CDK4 T172 phosphorylation we treated T47D breast cancer cells for 4 or 24 h with CDK4/6i and quantified CDK4 T172 phosphorylation by LC-MS/MS. Cyclin D1 was immunoprecipitated from cellular extracts and bound CDK4 was measured (Supplementary Figure 7). We then measuredCDK4 T172 phosphorylation within the complex and observed that 60–80% of total CDK4 was phosphorylated in both control (vehicle) and abemaciclib-treated cells (Fig. 3a). Similar CDK4 T172 phosphorylation levels were observed following palbociclib treatment. These data demonstrate that treatment with CDK4/6 inhibitors does not deplete CDK4 T172 phosphorylation. This contrasts regulation of kinase activity by autophosphorylation observed for CDK2, where CDK2 inhibition reduced T160 phosphorylation (active T-loop)^26^.

We next further investigated the regulation of CDK4 T172 phosphorylation following treatment with abemaciclib. Mitogen deprivation has been shown to decrease CDK4 activation through downregulation of CDK-activating kinases responsible for CDK4 T172 phosphorylation^24,27^. In mitogen-deprived cells, T172 phosphorylation was acutely decreased (Fig. 3b), consistent with previous observations^27^. However, mitogen deprivation combined with either abemaciclib or palbociclib treatment, showed sustained CDK4 T172 phosphorylation compared to control regardless of serum condition. These data indicate that CDK4/6 inhibitors bind and stabilize primed (active, but not operative) CDK4 pT172-cyclin D complex. We next compared alternative treatment regimen and observed that serum replacement (Treatment B) was able to increase T172 phosphorylation in the vehicle-treated condition while pT172 levels remained unchanged in the CDK4/6i treatment.

Given that abemaciclib stabilized active CDK4 pT172-cyclin D complex, and CDK4/6i are dosed at different frequencies clinically^4,5^, we next investigated whether sustained versus intermittent treatment with CDK4/6i was mechanistically required to maintain CDK4 signaling inhibition. We first measured Rb phosphorylation, a direct downstream target of CDK4, in the presence and absence of mitogenic signals and CDK4/6i. Removal of mitogenic signal (serum deprivation, 0% FBS) resulted in decreased Rb phosphorylation, with 54% pRb positive cells compared to 70% in complete serum condition (Fig. 3c). This was consistent with decreased CDK4-pT172, previously demonstrated (Fig. 3b). Additionally, treatment of cells with CDK4/6i decreased Rb phosphorylation, regardless of serum condition. When cells were allowed to recover in the absence of CDK4/6i, following serum replacement by cell wash out, pRb levels rapidly rebounded (Fig. 3d). This rebound was more pronounced in palbociclib-treated cells at equimolar concentrations.

### CDK4/6 inhibitor selectively mediates p21 dissociation from CDK4-cyclin D complex, but not p27

**Fig. 4:**
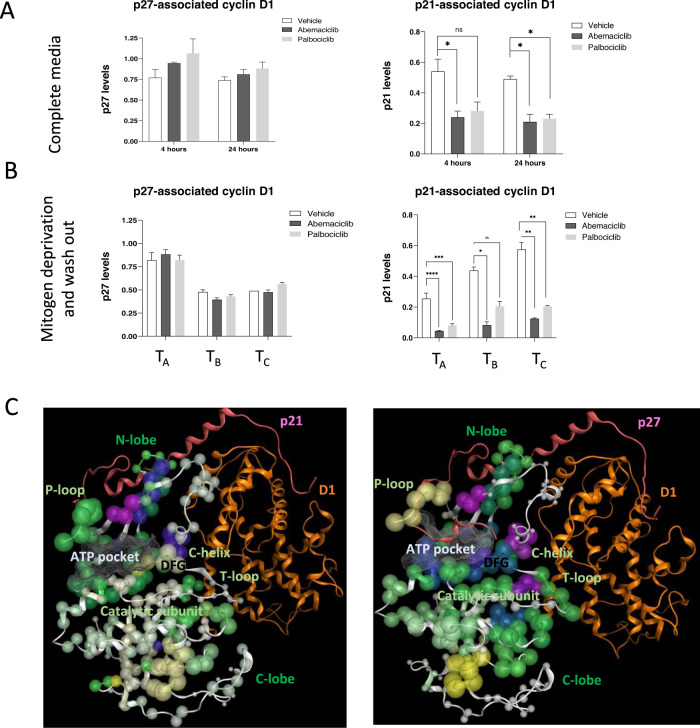
CDK4/6 inhibitors selectively mediate p21 dissociation from CDK4-Cyclin D complex. T47D cells were treated with CDK4/6 inhibitor in **A** complete media (*n* = 2) or **B** media lacking serum (*n* = 4). Cyclin D complexes were enriched and analyzed by LC-MS/MS. Quantification of cyclin D1-associated p21 and p27 are shown. Data shown was normalized to cyclin D1 levels. **B** Treatment regimen was as in Fig. 3b. **C** p21 (left) and p27 (right) HDX log10(k) projected on their corresponding models. p21/p27 bound to CDK4-D1 active complex are modeled in both cases using the most suitable structure 1jsu.pdb (active complex of CDK2-Cyc bound to p27) as template and mapping CDK4 and cyclin D1 sequence in their corresponding proteins. *p < 0.01, **p < 0.001, ***p < 0.0001, ****p < 0.00001, ns – not significant. P-values determined by pairwise t-test. Error bars represent ± standard error.

In addition to CDK4-T172 phosphorylation, CIP/KIP family proteins including p27 and p21 bind CDK4-cyclin D complex and are critical regulators of CDK4 activity^17,18^, though p21 and p27 response to CDK4/6i remains less understood^11^. We examined the interaction of endogenous p27 and p21 with CDK4-cyclin D complex in cells (T47D) by IP-MS, as in Fig. 3a. Regardless of mitogenic signals (serum conditions), p21 association with CDK4-cyclin D complex decreased following abemaciclib treatment compared to their relative control (Fig. 4A, B). By contrast, we observed no change in p27 association with CDK4-cyclin D in response to abemaciclib treatment compared to control.

To gain further insight into the binding of p21 and p27 to CDK4-cyclin D complex in response to abemaciclib treatment, we performed HDX-MS. We first examined pT172 CDK4-cyclin D1 in the presence of either p21 or p27 alone (Fig. 4C). Binding of p27 resulted in significantly increased protection of the CDK4 C-lobe, DFG and T-loop regions and high deprotection of the P-loop, relative to the ‘apo’ form (Fig. 4C, Supplementary Figure 8). Binding of p21 to active CDK4-pT172 complex resulted in minimal conformational changes in the C-lobe, and strong protection of the P-loop region. Additionally, we confirmed the inhibitory activity of p21 and p27 towards CDK4-cyclin D1 by in vitro kinase assay (Supplementary Figure 9). These results suggest that p21 and p27 have distinct modes of interaction with pT172 CDK4-cyclin D complex.

Given our structural and biological data (Fig. 4, Supplementary Figure 8 and 9), we examined whether p27 and p21 compete with abemaciclib for binding to CDK4. To test this, either p27 or p21 and abemaciclib were added sequentially to CDK4-cyclin D complex and the resultant interaction measured by HDX-MS. Addition of abemaciclib and then p27 to CDK4-cyclin D1 complex showed increased protection of the T-loop and deprotection of the hinge region and P-loop, compared to abemaciclib alone. The reciprocal experiment resulted in no detectable HDX effect in the ATP pocket regions (gray color, Supplementary Figure 10). In contrast, addition of abemaciclib then p21 showed increased mobility of CDK4 residues, denoting p21-mediated displacement of abemaciclib. In reciprocal HDX experiments, we observed increased protection of the hinge region, P-loop and C-lobe compared to p21 alone. Taken together, these HDX results suggest that of several possible explanations, the simplest is that both p21 and p27 effectively prevent abemaciclib binding to CDK4-cyclin D complex. However, abemaciclib is only able to efficiently outcompete p21 association with cyclin D1 from the CDK4-cyclin D complex at the concentrations and on the timescale of these experiments. Given the inhibitory role of p21 for CDK4-cyclin D, these results are consistent with our observation that abemaciclib stabilizes the active CDK4-cyclin D complex.

### Abemaciclib has a distinct mode of action on CDK4-cyclin D and CDK6-cyclin D complexes

**Fig. 5:**
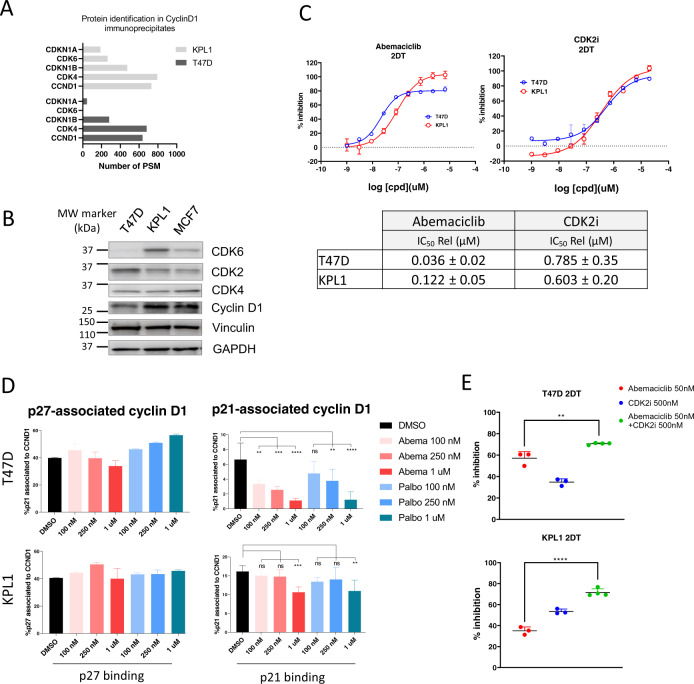
p21 competition in response to CDK4/6 inhibition and combination with CDK2 inhibitor in models of high or low CDK6 expression. **a** Protein identification by mass spectrometry of cyclin D1-associated proteins in CDK4/6 inhibitor sensitive (T47D) and resistant (KPL1) cells. **b** Western blot analysis of indicated proteins and (**c**) cell proliferation following CDK4/6 inhibitor and CDK2 inhibitor (PF-07104091) treatment in T47D and KPL1 cell lines. Representative IC50 dose-response curve is shown, table summarizes average of replicates (*n* = 4). **d** T47D and KPL1 cells were treated with vehicle or increasing concentrations of CDK4/6 inhibitors, as indicated by the x-axis, and p21 association with cyclin D1 measured. Average of two day-replicates and two technical replicates is shown. **e** T47D and KPL1 cells were treated for 48 h with abemaciclib or CDK2 inhibitor alone and combination and cell viability measured. **p < 0.01, ****p < 0.0001, ns – not significant. P-values determined by pairwise t-test. Error bars represent ± standard error.

Our data demonstrate that p21 is displaced from CDK4-cyclin D complex following treatment with abemaciclib. Recent studies describing mechanisms of resistance to CDK4/6i demonstrated that cells with increased CDK6-cyclin D levels compared to CDK4-cyclin D, are less responsive to CDK4/6i^20,28,29^, and decreased p21 displacement from CDK6-cylinD complex by CDK4/6i was also observed^20^. As such, we investigated whether abemaciclib-mediated p21 displacement was obligate for abemaciclib efficacy. We compared two breast cancer cell lines, the T47D and KPL1 models, the latter reported to be less responsive to CDK4/6i (*28*). In line with previous observations, we measured greater cyclin D1-CDK6 interaction and CDK6 levels in total cell lysate from KPL1 cells compared to the T47D cell line (Fig. 5a, b, Supplementary Table 3). We then treated cells with abemaciclib and measured cell viability and proliferation. We confirmed that T47D cells were more sensitive to abemaciclib inhibition compared to KPL1 cells (IC_50_: 36 nM vs. 122 nM) (Fig. 5c).

We next examined cyclin D1 interaction with p21 and p27 in CDK4/6 inhibitor-responsive and less-responsive cell models following treatment with abemaciclib and palbociclib. As in prior experiments, we observed a dose-dependent decrease in p21-cyclin D1 interaction in T47D cells (Fig. 5d). In KPL1 cells this response was less pronounced and regardless of cell sensitivity to inhibition, CDK4/6 inhibitors did not alter p27-cyclin D1 interaction (Fig. 5d). These results suggest that p21 displacement may be integral in mediating CDK4/6i efficacy, consistent with previous reports^20^. To this point, CIP/KIP proteins not only bind and inhibit CDK4 activity, but also CDK2 when in complex with cyclins^1,30^. To investigate the relationship between abemaciclib-mediated p21 displacement and subsequent CDK2 inhibition and overall efficacy we measured the sensitivity of T47D and KPL1 cells to abemaciclib or CDK2 inhibitor (PF-07104091) alone and in combination. In both cell models tested, the combination decreased cell survival compared to either single agent (Fig. 5e). This effect was greater in KPL1 cells, compared to single agent abemaciclib treatment. Overall, these data suggest that catalytic inhibition of CDK4 is critical for CDK4/6i efficacy, as well as p21 displacement from cyclin D1 and subsequent CDK2 inhibition^20^.

## Discussion

Previous attempts to crystalize CDK4-cyclin D have resulted in inactive structures which largely disrupt the cyclin N-terminus and the activation segment and contain active sites not competent for inhibitor or substrate binding. Here, we determined the first active CDK4-cyclin D3 structure, in complex with abemaciclib. Critical features of this active state include phosphorylated T172, an “in” αC-helix conformation, an extensive interface between the kinase and cyclin, and an ordered kinase activation segment that is buttressed by the cyclin N-terminus (the latter also uniquely ordered relative to the public structures) (Supplementary Movie 1). We hypothesize that the interaction of the CDK4 activation segment with the cyclin N-terminus may be critical to promote transition of CDK4 from the inactive to active conformation. Interestingly, residues 1–10 of the cyclin N-terminus remain disordered in the structure described here. This region of cyclin D contains the LXCXE sequence motif (residues 5–9) required for Rb recruitment^22^. It may be that these residues only become ordered in the context of the full catalytic complex: when ATP and Rb are bound^22^. Importantly, we demonstrate for the first time that wildtype phosphorylated CDK4 (pT172), bound to cyclin, can adopt an active confirmation analogous to that observed for other cyclin-bound CDKs (e.g. CDK2 and CDK6)^16^. Although pT172 and a native cyclin N-terminus may have been key for obtaining the active state, the use of a potent inhibitor (abemaciclib versus AMP-PNP), and differences in crystal packing may have also played a role. In addition, while the crystal contacts do not suggest an obvious stabilization mechanism for the active state, it cannot be definitively ruled out.

Beyond the extensive interaction of CDK4-cyclin D3 N-terminus described here, regulation of CDK4-T172 phosphorylation is required for proper CDK4 activity and its role as a biomarker for CDK4/6 inhibitor sensitivity has been previously hypothesized^15^. Nevertheless, the response of T172 phosphorylation to CDK4/6 inhibitors is not well understood^27^. Our structural, HDX and biological data demonstrates that palbociclib and abemaciclib bind and stabilize primed CDK4-pT172. This may have clinical implications, suggesting sustained treatment with CDK4/6 inhibitors may be required to maintain effective CDK4 inhibition. To support this hypothesis, we demonstrate that abemaciclib reduced pRb levels and maintained cell cycle inhibition, and withdrawal resulted in rapid rebound Rb phosphorylation and activation of CDK4 signaling.

Regulation of CDK4 is multifaceted and dependent on interaction with several protein partners^20,31^. We developed an IP-MS method to study the interaction of CDK4-cyclin D complexes in the presence of p21, p27 and CDK4/6. This system allows for the measurement of the physiologic cyclin D1 interactome and does not rely on exogenous expression of the protein components for observation. Consistent with a recent report describing the interaction between CDK4-cyclin D with p21 and p27 using a FRAP-based method, we show that CDK4-cyclin D-p21/p27 complexes formed regardless of CDK4/6 inhibitor treatment and CDK4/6 inhibition displaced p21, but not p27, from CDK4-cyclin D complexes^11,20^. Additional HDX-MS structural experiments corroborate our biological finding that p27 outcompetes abemaciclib for binding to CDK4-cyclin D complex.

Although our proposed mechanism of abemaciclib efficacy focused on CDK4 inhibition, it is well-described that not only do CIP/KIP proteins bind and inhibit CDK4, but also CDK2^20,30^. Since we show that abemaciclib mediates p21 displacement from CDK4-cyclin D complex, we hypothesized that displaced p21 and concomitant loss of CDK2 activity was critical for abemaciclib efficacy. In cell models of reduced CDK4/6 inhibitor sensitivity, abemaciclib was unable to displace p21 from CDK6-cyclin D and inhibit cell proliferation. However, treatment with combination abemaciclib and CDK2 inhibitor decreased cell viability to a greater extent. Further mechanistic studies are needed to better understand the relationship between CDK4/6 and CDK2.

In conclusion, our study 1) elucidates the first crystal structure of primed CDK4-cyclin D3 with native pT172 and bound abemaciclib, allowing for rational design of novel inhibitors which mechanistically target the CDK4/6 enzymes. 2) Demonstrates that biomarker identification for sensitivity to CDK4/6 inhibitors should be studied based on the composition of the complex, given the differential response to the inhibitors based on the presence of CDK4, CDK6, p21 or p27. 3) Suggests a unique model of CDK4/6 inhibitor-mediated stabilization of primed (active, but not operative) CDK4 pT172-cyclin D complex and the rapid reversal and rebound of pathway inhibition following abemaciclib removal (Supplementary Figure 11) further highlights the therapeutic benefit of sustained CDK4 inhibition.

## Methods

### Protein expression and purification

The gene for human CDK4 (RefSeq NM_000075), corresponding to amino acids 1–303, was TOPO-cloned into a custom pFastBac vector (ThermoFisher) in-frame with a C-terminal hexahistidine affinity tag. A similar (untagged) expression vector was constructed for human Cyclin D3 (RefSeq NM_001287434), corresponding to amino acids 1–259. A modified version of the Bac-to-Bac Baculovirus Expression System protocol (ThermoFisher), in combination with the DH10EMBacY bacmid (Geneva Biotech), was used to generate virus for infection of Sf9 cells. Equal volumes of virus for the two constructs were used to infect Sf9 that were then grown in 12 L of Sf-900 II SFM media for 48 h. Cells were harvested by centrifugation at 6,238 g for 11 min (Beckman Coulter Avanti J-26 XPI), and then frozen at −80 °C. After thawing, cell lysate (Lysis buffer: 20 mM Tris-HCl, pH 8.0, 500 mM NaCl, 10% glycerol, 25 mM Imidazole, 0.1% octyl beta-D-glucopyranoside (BOG), 5 mM dithiothreitol (DTT); Halt™ Protease Inhibitor Cocktail (1:200)) was clarified by centrifugation at 38,400 g for 30 min (Beckman Coulter Avanti J-26 XPI) and the supernatant passed over a Ni-NTA Agarose column (ThermoFisher). Following extensive application of wash buffer (20 mM Tris-HCl pH 8.0, 50 mM imidazole, 500 mM sodium chloride, 0.1% BOG, 5 mM DTT), the protein complex was eluted with buffer composed of 20 mM Tris-HCl pH 8.0, 250 mM imidazole, 500 mM sodium chloride, 0.1% BOG, 5 mM DTT and 10% glycerol. The protein was then subjected to a final gel filtration step (using HiLoad 16/60 superdex S200) in 10 mM HEPES pH 7.5, 150 mM sodium chloride, 10% glycerol, 0.1% BOG, and 5 mM DTT. Pooled fractions were concentrated to 14–20 mg/mL for crystallization experiments. Electrospray mass spectrometry (conducted on a Waters Xevo qTOF-MS) was used to confirm that the CDK4 protein was predominantly a singly phosphorylated species.

In vitro dephosphorylation with Lambda Protein Phosphatase (NEB #P0753, New England Biolabs), was performed according to the manufacturer’s instructions.

Briefly, 1 mM MnCl_2_ (Manganese Chloride) was added to the protein sample and 1000 units lambda phosphatase was incubated per 1 mg protein at 4 C overnight or until confirmed dephosphorylated by MS/ESI.

### Protein crystallization

Concentrated CDK4-Cyclin D3 in 10 mM Hepes pH7.5, 150 mM NaCl, 10% glycerol, 0.1% B-octylglucopyranoside, 5 mM DTT was co-crystallized with abemaciclib using the sitting-drop vapor-diffusion method in a 24-well tray at 21 °C. Crystals were obtained by mixing 3 μL of protein solution (with 0.25 mM abemaciclib and 5 mM TCEP, pH 7, included immediately before setup) with 1.5 reservoir solution (100 mM MES pH 6.5, 10% PEG 8000, 200 mM sodium acetate, and 10 mM TCEP pH 7). Seed crystals from a previous setup were included, although this may not have been crucial in obtaining large crystals. After 3 days, single rod-shaped crystals were harvested into cryoprotectant (20% glycerol + reservoir) and flash-frozen in liquid nitrogen.

### X-ray data collection and structure determination

Synchrotron X-ray diffraction data were collected on a single crystal at the Advanced Photon Source on beamline 31-ID-D (LRL-CAT). The resulting data were integrated using autoPROC^32^, and merged and scaled with AIMLESS^33^. The structure was solved by molecular replacement with PHASER^34^, using a search model derived from the structures of a CDK6-V-cyclin complex and the inactive CDK4-cyclin D3 complex (2F2C and 3G33, respectively)^13,23^. Specifically, this hybrid model was constructed by aligning and replacing the V-cyclin of the CDK6-V-cyclin complex with the cyclin D3 from the CDK4-Cyclin D3 complex. V-cyclin binding of CDK6 has the noteworthy effect of driving CDK6 into an activated state, even in the absence of phosphorylation of the activation segment^23^, so this model represented a reasonable approximation of the active CDK4-Cyclin D3 complex. Following successful molecular replacement, several rounds of model-building and refinement were conducted with the COOT^35^, BUSTER^36^, and Phenix^37,38^ packages. The final model was validated with MolProbity^39^. Additional data collection and refinement details can be found in Supplementary Table 1.

### Hydrogen deuterium-exchange mass spectrometry (HDX-MS) methods

Hydrogen deuterium-exchange mass spectrometry (HDX-MS) experiments were performed with an in-house automated modular system interfaced with an Orbitrap Q-Exactive mass spectrometer as previously described^40^. CDK4-cyclin D1 (phosphorylated and unphosphorylated) proteins were pre-incubated with abemaciclib at a 1:10 molar ratio (1 h at room temperature) in 50 mM Hepes buffer containing 50 mM MgCl_2_ and 1 mM od DTT (adjusted to pH 7.4) and diluted into D_2_O buffer of an equivalent composition. The CDK4-cyclin D1 (phosphorylated) experiments with p21 and p27 were prepared in the same manner but at 1:1 molar ratio. The on-exchange reactions were carried out by a 5-fold dilution of a 10 µM protein stock solution (with and without abemaciclib, p21 or p27) in the corresponding D_2_O buffer. Samples were prepared with three replicates of the following exchange times: D_min_, 10 s, 30 s, 90 s, 270 s, 810 s, 2430 s, 7290 s and D_max_. Following this on-exchange, samples were quenched with 320 mM TCEP containing 100 mM NaH_2_PO_4_ solution (pH 2.4) at 4 °C and kept at −80 °C until use. Samples were thawed and injected into the LEAP injection port following on-line pepsin digestion. The resulting proteolytic peptide mixtures were desalted on a 2 mm × 2 cm C8 trap column and eluted through a 10 cm × 2.1 mm C18 analytical column into the mass spectrometer (Q-Exactive, Thermo Scientific). HDX-MS data were processed with HDX Workbench data software^41^. Eight comprehensive csv files with the full set of HDX data are provided: 10.6084/m9.figshare.17748092.

All figures were built by rendering the results of each corresponding processed HDX dataset on the corresponding pdb or model using 2020.09 release of Chemical Computing Group’s Molecular Operating Environment (MOE) software- https://www.chemcomp.com/.

### Cell treatments

T47D cells were grown in 175 mL flasks in RPMI cell growth media supplemented with 0.2 Units/ml bovine insulin, fetal bovine serum (FBS) to a final concentration of 10%, and penicillin/streptomycin. Specific treatment regimens are described for each experiment. Briefly, T47D cells were treated in standard growth condition (10% FBS, insulin) or in serum starving medium (0% FBS, no insulin) for specific times with either DMSO, 1 μM of CDK4/6 compounds (abemaciclib or palbociclib). Following the 3 days treatment, cells were collected or washed in ice cold PBS twice and medium replaced for fresh medium containing 10% FBS and insulin supplement, in absence of compounds. Cells were washed in ice cold PBS and cell pellets were collected by trypsinization at specified time points. Cell pellets were snap frozen and stored at −80 °C.

### Cyclin D1 Immunoprecipitation

Cell pellets were lysed in 1 mL lysis buffer (20 mM Tris, 0.15 M NaCl, 1 mM EDTA, 1 mM EGTA, 1% Triton X-100, and 1X EDTA-free Halt protease & phosphatase inhibitor cocktail, pH 8.0). Following cell debris removal, protein concentrations were determined using the Bradford assay with bovine serum albumin as the standard. For each IP condition, T47D cell lysates at 2 mg were incubated with the specified antibody (Supplementary Table 4) overnight at 4 °C. Immunocomplexes were captured with protein A/G magnetic beads (Dynabeads, Thermo) 1 h at 4 °C. Microtubes were placed on a magnetic stand and un-bound fractions were collected (supernatants) and transferred to a clean tube as controls. Beads were washed with 1 mL lysis buffer twice followed by two washes in ice-cold phosphate buffered saline. Beads-bound protein complexes were stored in 40 μL of 20 mM Tris pH 7.4 at −20 °C until ready for mass spectrometric analyses. Initial assay optimization was performed comparing CDK4/cyclin D complex enrichment with specified antibodies (anti-cyclin D1 [Abcam, ab134175], anti-cyclin D3 [Cell Signaling Technology, 2936] or anti CDK4 [Abcam, ab68266]) compared to control condition with beads alone (Supplementary Table 4). Cyclin D1 immunoprecipitation provided best enrichment of CDK4-cyclin D1 complex (Supplementary Data 1). At least two independent replicates were generated per IP condition. Data was acquired in data-independent and targeted MS modes. Interacting protein partners (cyclin D1, CDK4 (Supplementary Figure 7), CDK2, p27, p21, and other known cyclin D interacting proteins) were quantified and protein abundance was measured and normalized to cyclin D1 levels.

### Sample preparation for mass spectrometric analyses

Immunocomplexes in magnetic beads were processed for tandem mass spectrometry essentially as described by Hale and collaborators using volatile reduction/alkylation conditions prior to trypsin digestion (Hale, 2004). Briefly, magnetic beads containing cyclin-D1 immunocomplexes were reduced and alkylated in 50 mM ammonium carbonate (MP biochemical), 1% iodoethanol (Sigma-Aldrich), 0.5% triethylphosphene (Sigma-Aldrich) and 50% acetonitrile (Burdick & Jackson), pH 11, for 1 hour at 37 °C. Samples were dried under high vacuum for at least 3 h prior to overnight digestion with 1 μg trypsin (Promega) in 100 mM Tris-HCl (Invitrogen), pH 7.5. Tryptic digests were extracted in 40 μL 20% acetonitrile (ThermoFisher Scientific Optima LC/MS grade), 20% acetic acid (Millipore AX0073–6) solution for 2 h and beads separated using 0.45 μm spin filters (Millipore UFC30HV00) prior to mass spectrometric analyses.

An Ultimate 3000 HPLC HPLC system was run at 250nL/minute with Solvent A as 0.1% formic acid in water (ThermoFisher Scientific Optima LC/MS grade) and Solvent B as 0.1% formic acid in acetonitrile (ThermoFisher Scientific Optima LC/MS grade). 10 μL of each sample was loaded onto a Thermo Acclaim pepmap 100 pre-column (75μm × 2 cm, C18, 3μm, 100 A; ThermoFisher Scientific) at a rate of 5 μl per minute. Pre-column wash conditions were 5 min with solvent A. Analytical column was the Thermo pepmap RSLC (75 mm × 15 cm, C18, 3 mM, 100 A; Thermofisher). Analytical column separation was at 250nL/minute with gradient of solvent B consisting of 2 to 40% in 50 min followed by 6 min at 80% solvent B. Michrom Bioresources 6 bovine protein tryptic digest standards (P/N PTD/00001/64) were analyzed as instrument performance controls.

A ThermoFinnigan Q-Exactive Orbitrap mass spectrometer was used with the Thermo Easy Spray source at +2400 volts. For data dependent mass spectrometric analyses, the acquisition was one parent MS scan in the FT mode (70,000 resolution) and 15 data dependent MS/MS scans collected. Ions with the following charge states were rejected on the instrument: unassigned, +1, 5–8, and >8. Dynamic exclusion was 20.0 s. For data-independent mass spectrometric analyses (PRM), the mass spectrometer was operated targeting peptide species as described in supplementary data (Supplementary Table 5). To assess CDK4 T172 phosphorylation we specifically monitored fragmentation spectra for the tryptic peptide (IYSYQMAL**T**_**172**_PVVVTLWYR) spanning this region in CDK4 Fig. 2B. We included stable isotope labeled (SIL) peptides, phosphorylated and un-phosphorylated, post digestion prior to mass spectrometric analyses following standard protocols. Mass spectrometric extracted ion chromatogram signals for the SIL forms of the CDK4 tryptic peptide were used to normalize signals from endogenous CDK4 T172 peptides, phosphorylated and unphosphorylated, and used to assess the role of CDK4 inhibition

Data analyses for data-dependent mass spectrometry data were using an in-house software suite with Sequest, X!Tandem, and Protein pilot search engines. Data-independent analyses for targeted peptides were with Pinpoint software.

### Target engagement assay with ATP/ADP probe

Target engagement assay using ATP and ADP desthiobiotin probes (ThermoFisher Scientific) was performed as described by vendor. Briefly, recombinant proteins (CDK4-CyclinD1) of 1 P or 0 P (PID information) were prepared at 1 μM final concentration in kinase reaction buffer and incubated with compounds at a specified concentration for 30 min. ActivX™ Desthiobiotin-ATP and ADP Probe mixture was prepared in reaction buffer (20 mM HEPES, 1 mM DTT, 5 mM MgCl_2_) and added to the recombinant protein preparation at 50 μM final concentration and incubated for another 30 min. Following the reaction, in-solution digestion was performed. Briefly, reaction buffer was adjusted to 25 mM Ammonium bicarbonate (25 mM) and protein was reduced and alkylated with 10 mM DTT and 50 mM IAM, respectively. Protein digestion was performed with trypsin sequencing grade modified trypsin (0.16 μg per sample) at 37 °C, overnight. Samples were dried in N_2_ and desalted using C18-zip-tips (Millipore) for MS analysis. Biotinylated peptides were not purified in order to estimate rate of modification to the unmodified part. Peptide sequence corresponding to modification of lysine 142 (DLK[+ 196.1]PENILVTSGGTVK) or non-modified (DLKPENILVTSGGTVK) was monitored by MRM using a 6460 Agilent triple quadrupole system. Average of the untreated samples was calculated and ratios to this control were measured for each condition to determine percent ATP/ADP probe binding. % binding = ((normalized AUC treated)/average(normalized AUC untreated)) × 100; % Inhibition = [1 – ((average treated signals)/average control signals))] × 100. Data analysis was carried out using Prism software v5.03. A nonlinear regression with a three-parameter fit was applied to estimate binding inhibition constants.

### High-content imaging cell-based assays

Rb phosphorylation at Serine 780 was monitored by high-content imaging. T47D breast cancer cells were serum starved for 72 h or maintained in complete media in the presence or absence of CDK4/6i (abemaciclib and palbociclib at 1 μM) and wash-out experiments were performed to monitor sustained response. Briefly, cells were fixed with paraformaldehyde and S-780 Rb phosphorylation was monitored with anti-Rb (pS780) antibody (Millipore); to track cell viability, cells were stained with propidium iodide (PI), which was used to establish the percentage of cells with pRb-S780 signal relative to the total PI-stained subpopulation. Plates were acquired using an Acumen Explorer eX3 (TTP Labtech). Assay was carried out as 4 independent biological replicates of 4 independent treatment replicates. Average of % pRB-S780 positive cells from replicate analyses is shown. Western blot was performed as we have previously described^42^. Unmodified gel images are presented in Supplementary Figure 12.

### Cell proliferation and combination study

Cell proliferation following abemaciclib and CDK2i (PF-07104091) treatment was measured by high-content imaging in T47D (ATCC; Cat #: HTB-133) and KPL1 (DSMZ; Cat #: ACC 317) cell lines. Cell lines tested negative for mycoplasma. 1000 cell/well were seeded in 384-well plate (Corning# 356663) in 20 μL of RPMI 1640 (Gibco, A10491), or DMEM (Sigma, D5796), respectively in 10% FBS at 37 °C, 5%CO_2_. Assay-ready plates containing 1:3 serial dilutions of compounds in 100% DMSO were prepared using acoustic dispensing technology (ECHO), final starting concentration was 20 µM. Compound-treated plates were incubated for 4 days (2 doubling times) and cell proliferation estimated using PI staining. For cell staining, cells were fixed with paraformaldehyde and permeabilized 96% cold Ethanol (70% final concentration). Then, stained with PI (Invitrogen # P3566) and 1:1000 RNAse (SIGMA# R6513, 50 mg/ml) was added to cells. Data was acquired using an Acumen Explorer Ex3 (TTP Labtech). Raw data were analyzed with Cellista software and normalized using a Staurosporin (internally synthesized) as a positive control for inhibition of cell proliferation and 0.3% DMSO as a negative control. Relative IC50 values were determined by curve fitting to a four-parameter logistic equation: Y = bottom + ((top-bottom)/ (1 + exp(HillSlope*(X – LnIC50)). Data were plotted using GraphPad Prism. As all studies were performed in cell line models, no ethical approval for animal models or human samples was sought.

### In vitro kinase assay

CDK4-cyclin D1 activity was measured by the phosphorylation of C-Terminal Retinoblastoma Fragment (CTRF) using [γ-^33^P] ATP at 20 °C. We compared the activity of the phosphorylated form of CDK4 bound to cyclinD1 and its modified un-phosphorylated version. Briefly, enzyme reactions were performed in 25 μL assay reaction volume in a 96-well plate filtration assay format. Kinase assay buffer contained 50 mM Hepes at pH 7.5, 5 mM MgCl_2_, 5 mM dithiothreitol (DTT), 0.002% (w/v) BSA and 0.01% TRITON X-100. 10 μL of CDK4-cyclin D1 enzyme complex (ProQinase, 0142-0143-1) or internal protein constructs (2 nM final concentration in the assay) was added onto a substrate mixture containing ATP at 0.04 mM (1.12 mCi [^33^P] –ATP per μmol ATP) (PerkinElmer #NEG602K250UC) and a constant concentration of 0.5μM C-Terminal Retinoblastoma Fragment (CTRF) was used (Upstate #12–439). Reagents were mixed and incubated for a time course up to 120 min at 20 °C. The reaction was terminated by the addition of 80 μL 10% (v/v) H_3_PO_4_ and precipitation of material onto glass fiber filter plates (Millipore, MAFCN0B50). The wells were washed 4 times with 0.5% H_3_PO_4_ and the radioactivity incorporated was measured with a microplate scintillation counter (Microbeta Trilux, Wallac).

### Statistical analysis

The one-way analysis of variance (ANOVA) was used to determine statistically significant differences between the means of abemaciclib, palbociclib and the control groups followed by the post-hoc pairwise t-test to compare the treated group and the control with JMP 15.0. Error bars represent ± standard error, unless otherwise noted.

## Supplementary information

Supplementary Information

Supplementary Movie 1

Supplementary Data 1

## Data Availability

All X-ray and HDX-MS data and materials are publicly available. Structure factors and coordinates for crystal structures are available in the Protein Data Bank under accession codes 7SJ3. X-ray data collection and refinement details are included within Supplemental Table 2. Eight comprehensive csv files with the full set of HDX data are provided (described in HDX-MS methods): 10.6084/m9.figshare.17748092. Respective peptides used for IP-MS are detailed in Supplemental Table 5.
