# Author Correction: Crystal structure of active CDK4-cyclin D and mechanistic basis for abemaciclib efficacy

**DOI:** 10.1038/s41523-022-00503-0

**Published:** 2022-12-28

**Authors:** Severine Isabelle Gharbi, Laura A. Pelletier, Alfonso Espada, Jesus Gutiérrez, Sonia Maria Gutiérrez Sanfeliciano, Charles T. Rauch, Maria Patricia Ganado, Carmen Baquero, Elisabet Zapatero, Aiping Zhang, Jordi Benach, Anna-Maria Russell, Leticia Cano, Sandra Gomez, Howard Broughton, Nicholas Pulliam, Carmen Maria Perez, Raquel Torres, Marjoke F. Debets, Alfonso de Dios, Oscar Puig, Mark T. Hilgers, Maria Jose Lallena

**Affiliations:** 1 Discovery Chemistry Research & Technology, Eli Lilly and Company, Madrid, Spain; 2 Lilly Biotechnology Center, Eli Lilly and Company, San Diego, CA USA; 3 Eli Lilly and Company, Indianapolis, IN USA; 4 Eli Lilly and Company, New York, NY USA

**Keywords:** Breast cancer, Tumour biomarkers

Correction to: *npj Breast Cancer* 10.1038/s41523-022-00494-y, published online 29 November 2022

In this article the wrong figures appeared as Fig. 2 and Fig. 5; the figures should have appeared as shown below.

Fig. 2 corrected
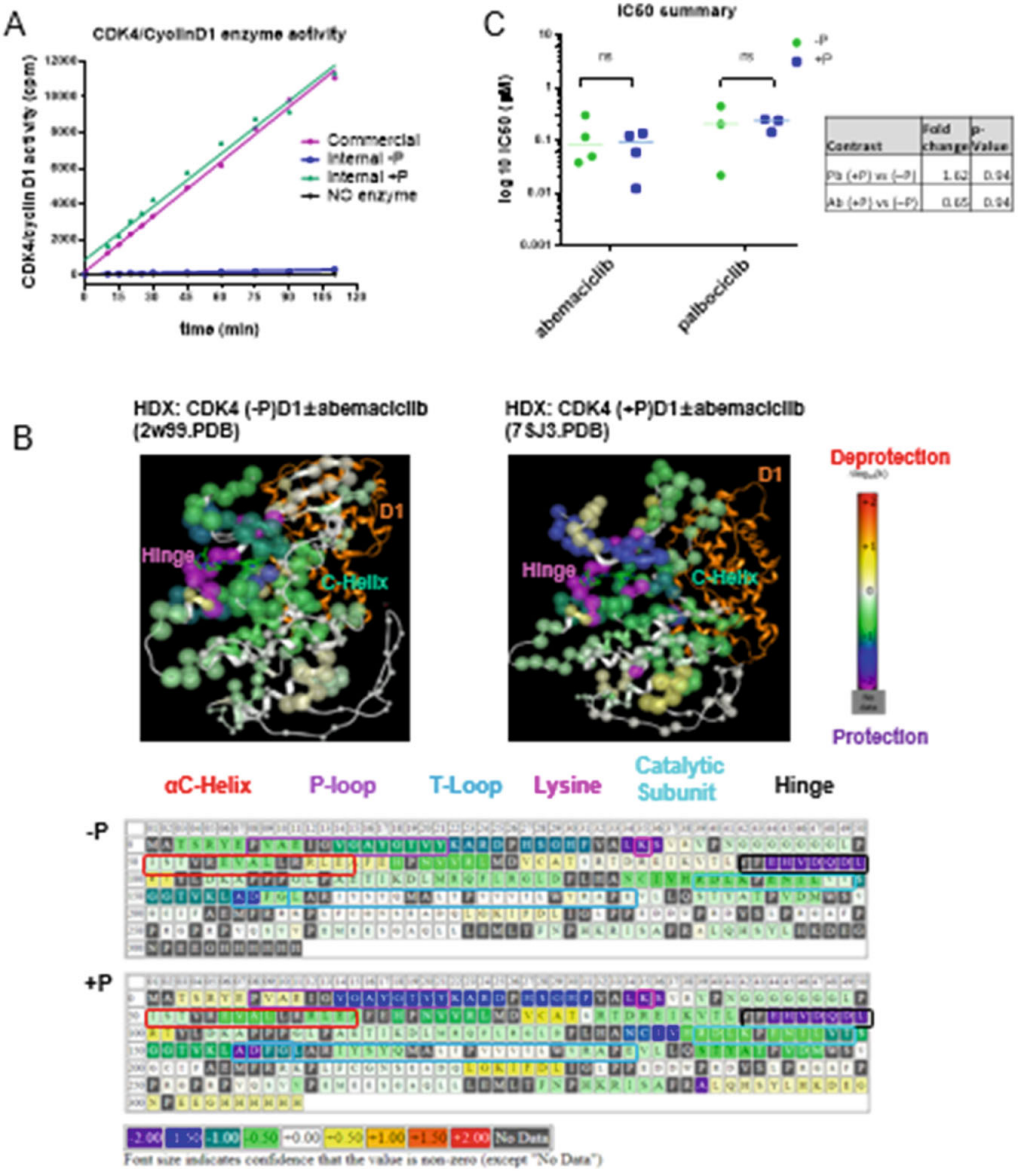


Fig. 5 corrected
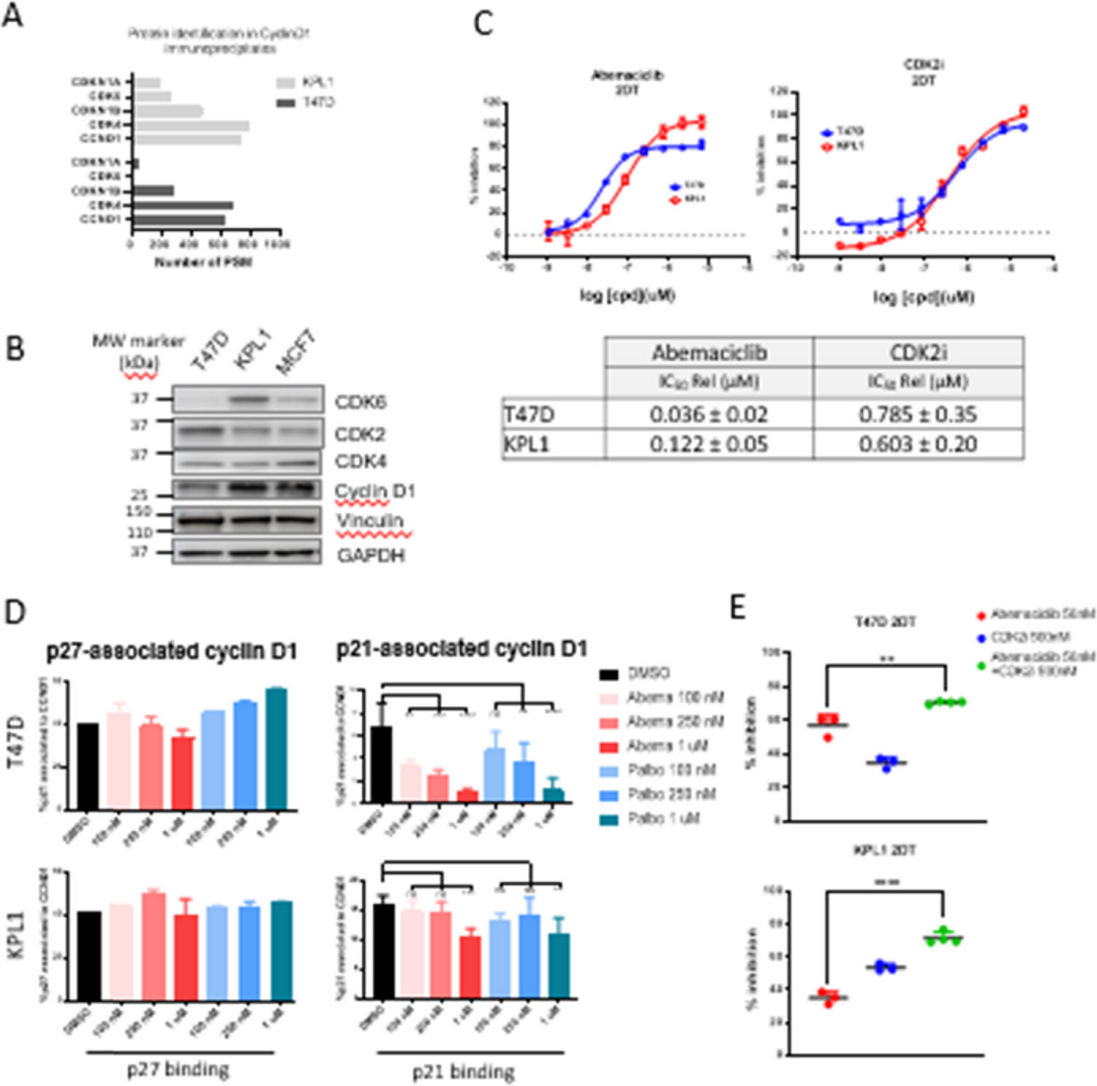


The original article has been corrected.

