# Supplementary material for: Crystal structure of active CDK4-cyclin D and mechanistic basis for abemaciclib efficacy
Source: NPJ Breast Cancer. 2022 Nov 29;8:126. doi: 10.1038/s41523-022-00494-y (PMC9709041; doi:10.1038/s41523-022-00494-y)
Supplement: Supplementary file 1 — Supplementary Information [file 41523_2022_494_MOESM1_ESM.pdf]

Supplementary Information:

Supplementary Figure 1: Electron density map showing phosphorylation at reside T172 (**a**), and an unambiguous conformation for abemaciclib in the ATP site (**b**). Both views are derived from a 2Fo-Fc composite simulated annealing omit map generated in Phenix, and contoured at 1.0σ.

Supplementary Figure 2: The transition of CDK4-cyclin D3 from the inactive to active state. **(a)**The Cyclin D undergoes a significant rotation and translation, relative to the kinase domain, upon activation. **(b)** This also involves a significant rotation and translation that yields a large increase in buried surface area between kinase and cyclin (3,292Å^2, compared to ~2,300Å^2 observed for the inactive structures), and a final heterodimer arrangement like that observed in previously described CDK-Cyclin structures.

Supplementary Figure 3: CDK4-cyclin D3 kinase activity. *in vitro* kinase assay with [γ-33P] ATP of active CDK4-cyclin D3 and CDK4-cyclin D1 (control) towards the C-Terminal Retinoblastoma Fragment (CTRF)

Supplementary Figure 4: Interactions of abemaciclib with the ATP site of CDK4-cyclin D3. The aminopyrimidine ring Hydrogen bonds with the backbone of residue V96. The pyridyl group binds through a water-mediated Hydrogen bond with H96. The free amine of the benzimidazole shares a Hydrogen bond with K35. The abemaciclib-CDK6 structure is shown superposed in orange (5L2S.pdb). Although the interactions are similar, the lack of Cyclin in the CDK6 structure results in a C-helix "out" conformation, and differences in the activation segment conformation.

Supplementary Figure 5: HDX-MS heatmaps of unphosphorylated and phosphorylated CDK4-cyclin D in the absence of abemaciclib. CDK4 domains are highlighted and the DFG motif is shown within the T-loop.

Supplementary Figure 6: HDX heatmaps of CDK4 alone and differential maps of CDK4-cyclin D single ligand experiments with either abemaciclib, p27 or p21, compared to CDK4 alone. CDK4 domains are highlighted, and the DFG motif is highlighted within the T-loop.

Supplementary Figure 7: Quantification of CDK4 associated with cyclin D1 following immunoprecipitation was monitored by LC-MS/MS. This data was generated from experiments reported in: (**a**) Figure 3A and 4A; (**b**) Figure 3B and 4B; (**c**) Figure 5D. Error bars represent ± standard error

Supplementary Figure 8: Projection of pT172 CDK4-cyclin D1-p21/p27 focused on ATP pocket. CDK4 domains are highlighted and the DFG motif is shown within the T-loop. More blue hues indicate increased protection, less flexibility. More red hues indicate less protection, increased flexibility.

Supplementary Figure 9: p21 and p27 inhibits the activity of CDK4 activity *in vitro*. Biochemical analysis of the effect of p21 and p27 peptides in the kinase activity of CDK4/cyclinD1 recombinant protein.

Supplementary Figure 10: (a) Projection and **(b)** sequence and domain alignment HDX-MS differential maps of p21/p27 and abemaciclib sequential binding. CDK4 domains are highlighted and the DFG motif is shown within the T-loop. Bluer hues indicate increased protection, less flexibility. More red hues indicate decreased protection, greater flexibility. font color is chosen only for optimum contrast with the background.

Supplementary Figure 11: Proposed model differentiating the effects of continuous versus intermittent treatment with CDK4/6 inhibitors. (a) In the absence of CDK4/6

inhibitors active CDK4 pT172-cyclin D complex phosphorylates and inactivates Rb, allowing for the release of E2F family of transcriptional factors, cell cycle progression and tumorigenesis. **(b)** We suggest a novel model of CDK4/6 inhibitor-mediated stabilization of **(I)** primed (active, but not operative) CDK4 pT172-cyclin D complex, demonstrating **(II)** rapid reversal and rebound activation of the CDK4 signaling pathway following abemaciclib removal highlighting the benefits of (B. I) continuous versus (B. II) intermittent CDK4/6 inhibitor dosing. In B. II, the dashed line around p21 indicates that p21 may or may not be bound to complex following abemaciclib removal.

Supplementary Figure 12: Unmodified gel images related to Figure 5B.

Supplementary Table 1: Discussion of CDK4 structures published to date

Supplementary Table 2: Data collection and refinement for crystal structure determination

Supplementary Table 3: Cyclin D1-immune complex analyzed by LC-MS. Components of the complex are shown. Table denotes percent protein coverage, total number of peptides identified and number of peptide spectrum search (PSM) events for cyclin D1 (CCND1), CDK4, CDK6, p21 (CDKN1A) and p27 (CDKN1B).

Supplementary Table 4: List of antibodies used for western blot and immunoprecipitation

Supplementary Table 5: Targeted masses in the mass spectrometer for peptides in proteins part of the Cyclin D1-CDK4 complex. Table denotes amino acid position within the targeted protein, the amino acid sequence with modifications, either observed or as part of the standard isotope labels for peptide standards, the mass to charge ratio (m/z) and the charge state for the targeted peptides. Immunocomplexes were for cyclin D1 and targeted peptides were specific for Cyclin D1, CDK4, p27 and p21. Isotope labels are denoted as ^{13}C or ^{15}N in the amino acids containing isotope atoms.

Supplementary Data 1: Comparison of immunoprecipitation experiments with anti-CDK4, anti-cyclin D1 and anti-cyclin D3 antibodies compared to control immunoprecipitation with beads alone.

Supplementary Movie 1: Depiction of CDK4-cyclin D3 complex in the inactive and active state. Note: the movements do not represent the trajectory of transition from the inactive to active state but is intended to show the relationship between the two states.

Supplementary Table 1

PDB ID	Resolution, Å	R free	Space group	A,B,C (Å)	α,β,γ (˚)	Protein Complex	Key Mutations	Release date	Reference (PMID)
2W9Z	2.45	0.272	P 21 21 21	57.20, 65.01, 188.70	90, 90, 90	CDK4-cyclin D1	CDK4: T172A, 42-GGGGG → EE	2009	19237565
2W99	2.80	0.270	P 21 21 21	57.02, 64.68, 188.75	90, 90, 90	CDK4-cyclin D1	CDK4: T172A, 42-GGGGG → EE	2009	19237565
2W96	2.30	0.259	P 21 21 21	55.91, 64.69, 168.69	90, 90, 90	CDK4-cyclin D1	CDK4: T172D, 42-GGGGG → EE	2009	19237565
2W9F	2.85	0.300	P 21 21 21	58.00, 64.28, 187.62	90, 90, 90	CDK4-cyclin D1	CDK4: T172F, 42-GGGGG → EE	2009	19237565
3G33	3.00	0.314	P 42 21 21	141.30, 141.30, 143.53	90, 90, 90	CDK4-cyclin D3	Cyclin D: N-terminal FLAG tag	2009	19237555
5FWP	7.20	Cryo-electron microscopy				CDK4-HSP90-CDC37		2016	27339980
5FWL	9.00					CDK4-HSP90-CDC37		2016	27339980
5FWM	8.00					CDK4-HSP90-CDC37		2016	27339980
5FWK	3.90					CDK4-HSP90-CDC37		2016	27339980
6P8E	2.30	0.222	P 21 2 21	62.41, 67.49, 187.28	90, 90, 90	CDK4-Cyclin D1-p27		2019	31831640
6P8H	3.19	0.258	P 21 2 21	62.62, 67.98, 185.36	90, 90, 90	CDK4-Cyclin D1-p21		2019	31831640
6P8F	2.89	0.242	P 21 2 21	62.86, 66.63, 184.24	90, 90, 90	CDK4-Cyclin D1-p21		2019	31831640
6P8G	2.80	0.275	P 21 2 21	62.58, 66.73, 184.85	90, 90, 90	CDK4-Cyclin D1-p27		2019	31831640
7SJ3	2.73	0.253	P 65 2 2	117.80, 117.80, 170.33	90, 90, 120	CDK4-Cyclin D3		2021	Current Work

Supplementary Table 1

Discussion of CDK4 structures published to date. None of the previously described structures have exhibited the active kinase conformation. In the two most recent studies (9, 11), an inactive conformation was expected. The first of these yielded Cryo-EM structures of a partially unfolded kinase domain in complex with chaperones Hsp90 and Cdc37, with the kinase domain N-terminal domain disordered (11). The other was a crystallographic study of CDK4-Cyclin D1 bound to p21 and p27 (9). In this case, by analogy with structural studies of CDK2-Cyclin A, it was anticipated that binding of p27/p21 would disrupt the catalytically active conformation of the kinase through the interactions of its D2 domain with the kinase N-terminal lobe, and this was indeed found to be the case.

The reasons that the structures reported in 2009 were inactive is more nuanced (8,10). The structure determined by Takaki et al. (3G33) was found to lack any electron density near T172 that would be consistent with phosphorylation, despite LC-MS results that indicated that their crystallization sample was ~62% phosphorylated at this residue (10). Additional experiments demonstrated that the protein in the crystals showed no evidence of phosphorylation, thereby suggesting that the mother liquor preferentially induced the crystallization of only the non-phosphorylated species out of the mixed solution, yielding an inactive structure. The protein used in our crystallization experiments was ~81% phosphorylated at T172 and did not display this sort of biased crystallization (see Methods). Given the probable importance of the N-terminus of Cyclin in the formation of active complex, it is also possible that the FLAG-tagged Cyclin D3 construct used in crystallization of 3G33 was not ideal. Although this affinity tag was fused immediately upstream of the LXCXE motif, it may have had a negative steric or electrostatic effect on Cyclin-CDK4 interactions in the immediate vicinity.

The four structures reported by Day et al. were more highly engineered, including point mutations at T172 (8). Two are “phospho knockouts” with T172A substitutions (2W9Z and 2W99) and show the low kinase activity that would be expected (in addition to the inactive conformation). Another construct (2W96) included a phosphomimetic mutation, T172D. However, the very low level of enzyme activity observed indicates that it did not mimic the active pT172 state, and indeed, the conformation of the activation segment is comparable to that of other inactive structures. The fourth structure described in this report is purported to be both Wildtype and phosphorylated at T172 (2W9F). However, the activation segment surrounding T172 was found to be disordered (residues 171-176). Further, the header of the coordinate file and additional data for this entry at the Protein Databank (rcsb.org), suggest that this structure is not WT but has a T172F mutation. This non-conservative (possibly disruptive) mutation might explain the activation segment disorder. Of note, the other structures lack this disorder.

Supplementary Table 2

**Data Collection**	
Space group	$P6_522$
Cell dimensions	
a, b, c (Å)	117.65, 117.65, 170.27
α, β, γ (°)	90, 90, 120
Resolution (Å)	19.78– 2.51 (2.65–2.51)*
$CC_{1/2}$	0.999 (0.824)
Mean((I)/sd(I))	21.5 (2.6)
R_{merge}	0.103 (1.567)*
R_{meas}	0.107 (1.634)*
Completeness (%)	99.7 (99.8)*
Multiplicity	21.6 (22.1)*

*** Highest resolution shell (10% of reflections)**

**Refinement**	
No. of reflections	24,285
R_{free}	0.2318
R_{work}	0.1983
No. of protein chains	2
No. of amino acids	520
No. of atoms (non-Hydrogen)	
Protein	4004
Water	45
Other	51
Mean B-factors	
Protein	59.1
Water	62.8
Other	68.8
RMS deviations	
Bond lengths (Å)	0.0059
Bond angles (°)	1.1862
Ramachandran plot (%)	
Favored	96.0
Allowed	99.2
Outliers	0.80

Supplementary Table 3

	T47D			KPL1		
Gene Symbol	Coverage [%]	# Peptides	# PSMs	Coverage [%]	# Peptides	# PSMs
CCND1	72	19	636	81	24	731
CDK4	55	15	680	67	20	793
CDKN1B	69	10	281	73	13	474
CDK6				56	16	265
CDKN1A	30	3	44	70	10	188

Supplementary Table 4

Antibody	Supplier name	Catalog number	Assay
**CDK4**	Cell Signaling Technology	12790	WB
**CDK4**	Abcam	ab68266	IP
**Cyclin D1**	Abcam	ab134175	WB/IP
**Cyclin D3**	Cell Signaling Technology	2936	IP
**CDK6**	Abcam	ab124821	WB
**CDK2**	Cell Signaling Technology	2546	WB
**GAPDH**	Cell Signaling Technology	97166	WB
**Vinculin**	Sigma	V9131	WB

Supplementary Table 5

	CDK4 Peptides	m/z	Charge
**CDK4_39-55**	VPN[Deamid]GGGGGGGLPISTVR	748.4	2
**CDK4_140-155**	DLKPENILVTSGGTVK	557.6	3
**CDK4_226-240**	IFDLIGLPPEDDWPR	594.9	3
**CDK4_164-181**	IYSYQMALTPVVVTLWYR	735.1	3
**CDK4_164-181_ox**	IYSYQM[Oxid]ALTPVVVTLWYR	740.4	3
**SIL CDK4_164-181**	IYSYQMA[^{13}C-^{15}N]LTPVVVT[^{13}C-^{15}N]LWYR	739.7	3
**SIL_CDK4_164-181_ox**	IYSYQM[Oxid]A[^{13}C-^{15}N]LTPVVVT[13C-15N]LWYR	745.1	3
**pCDK4_164-181**	IYSYQMALT[phos]PVVVTLWYR	761.7	3
**pCDK4_164-181_ox**	IYSYQM[Oxid]ALT[Phos]PVVVTLWYR	767.0	3
**SIL pCDK4_164-181**	IYSYQMA[^{13}C-^{15}N]LT[phos]PVVVT[^{13}C-^{15}N]LWYR	766.4	3
**SIL_pCDK4_164-181_ox**	IYSYQM[Oxid]A[^{13}C-^{15}N]LT[Phos]PVVVT[^{13}C-^{15}N]LWYR	771.7	3
	**cyclin D1 Peptides**		
**Cyclin D1_16-26**	AYPDANLLNDR	631.3	2
**Cyclin D1_88-95**	FLSLEPVK	466.8	2
**Cyclin D1_195-218**	FISNPPSMVAAGSVVAAVQGLNLR	800.1	3
	**p27 Peptides**		
**P27_31-43**	NLFGPVDHEELTR	509.6	3
**P27_74-81**	YEWQEVEK	555.8	2
**P27_170-189**	TEENVSDGSPNAGSVEQTPK	1023.5	2
	**p21 Peptides**		
**P21_21-32**	LFGPVDSEQLSR	674.3	2
**P21_76-83**	LYLPTGPR	458.8	2

Supplementary Figure 1

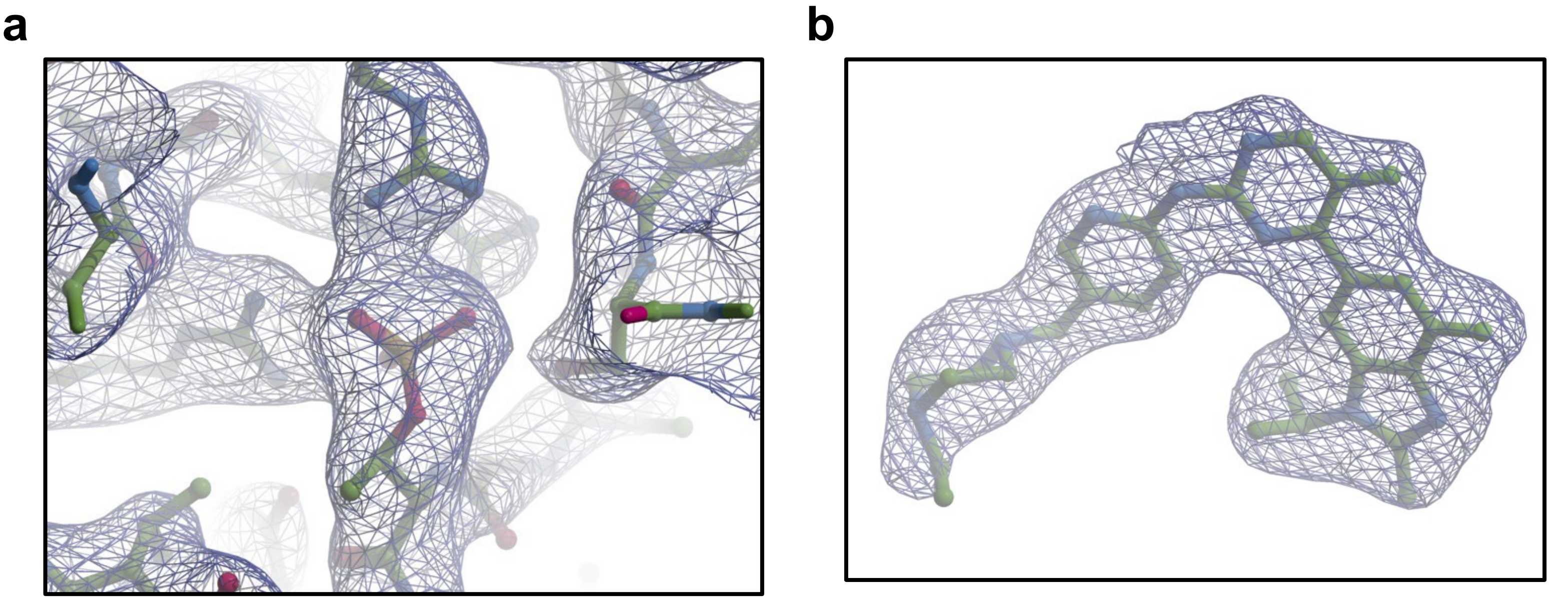

Supplementary Figure 1. Electron density map showing phosphorylation at reside T172 (**a**), and an unambiguous conformation for abemaciclib in the ATP site (**b**). Both views are derived from a 2Fo-Fc composite simulated annealing omit map generated in Phenix, and contoured at 1.0σ.

Supplementary Figure 2

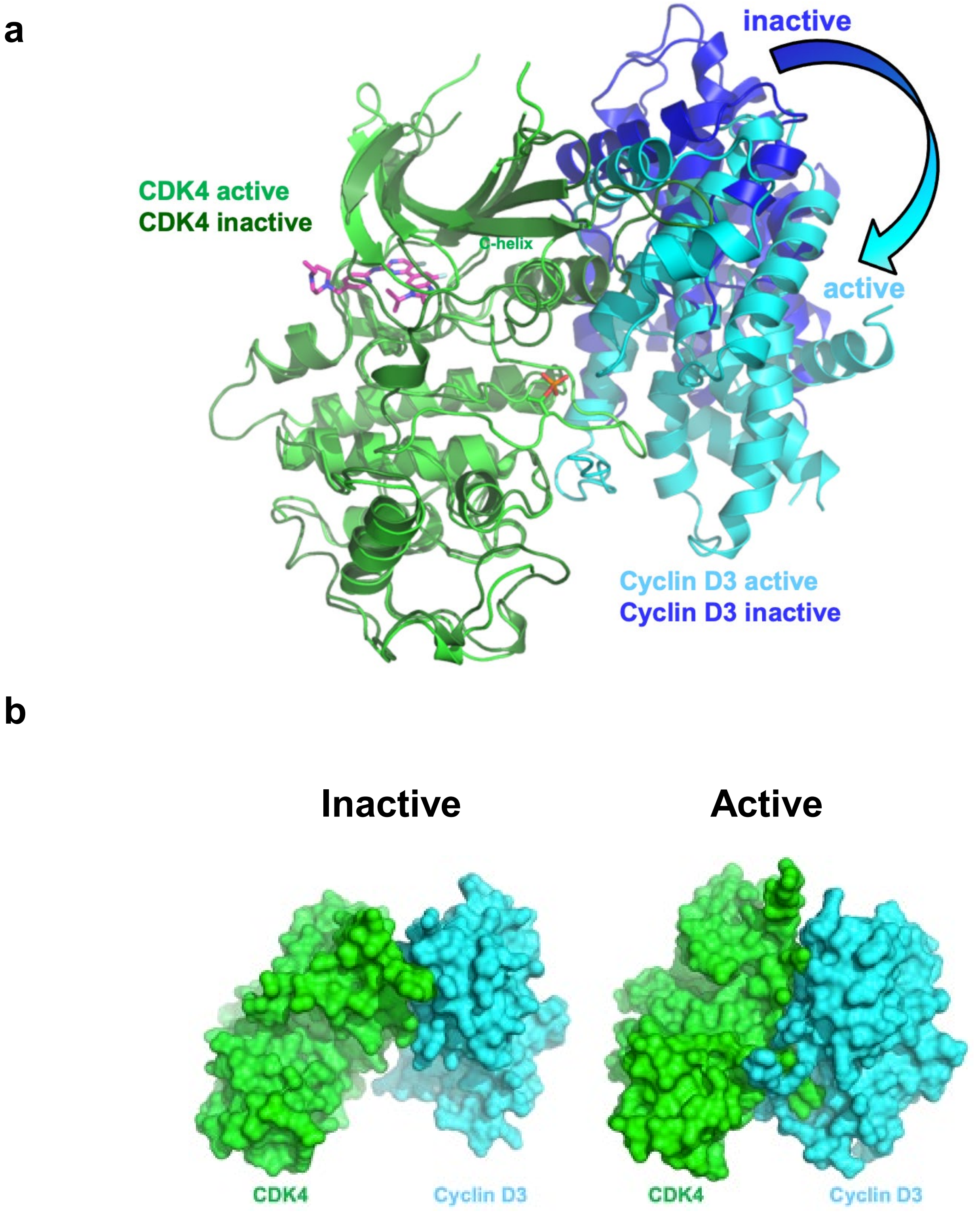

Supplementary Figure 2. The transition of CDK4-cyclin D3 from the inactive to active state. **(a)**The Cyclin D undergoes a significant rotation and translation, relative to the kinase domain, upon activation. **(b)** This also involves a significant rotation and translation that yields a large increase in buried surface area between kinase and cyclin (3,292 Å^2, compared to ~2,300 Å^2 observed for the inactive structures), and a final heterodimer arrangement like that observed in previously described CDK-Cyclin structures.

Supplementary Figure 3

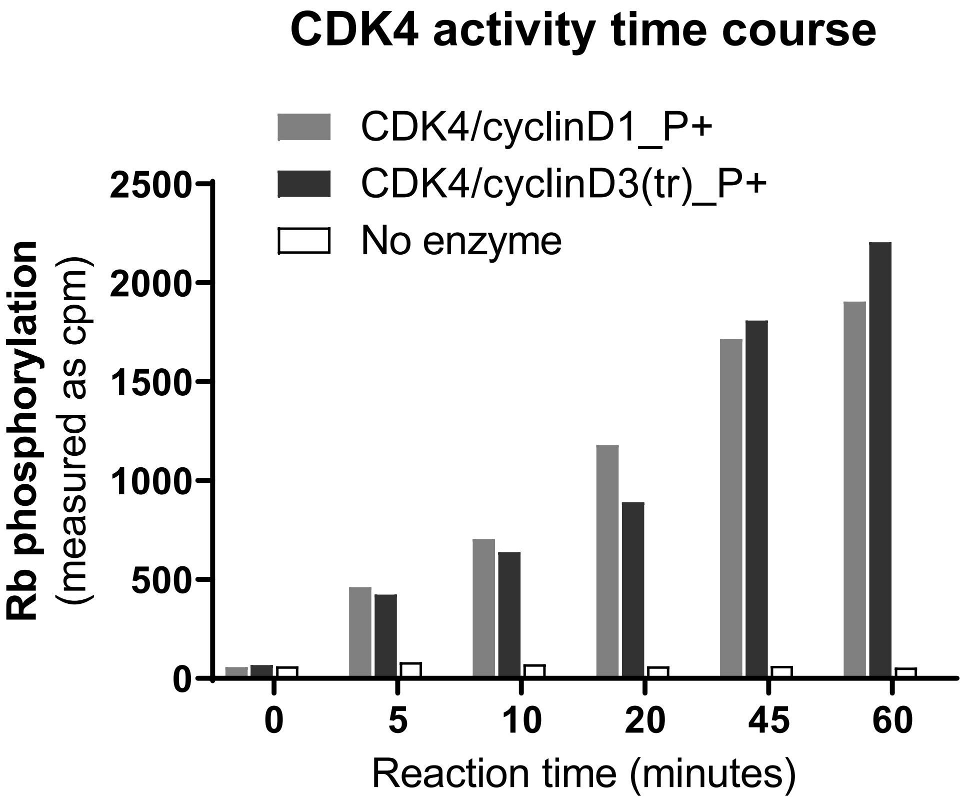

Supplementary Figure 3. CDK4-cyclin D3 kinase activity. *in vitro* kinase assay with [γ-33P] ATP of active CDK4-cyclin D3 and CDK4-cyclin D1 (control) towards the C-Terminal Retinoblastoma Fragment (CTRF)

Supplementary Figure 4

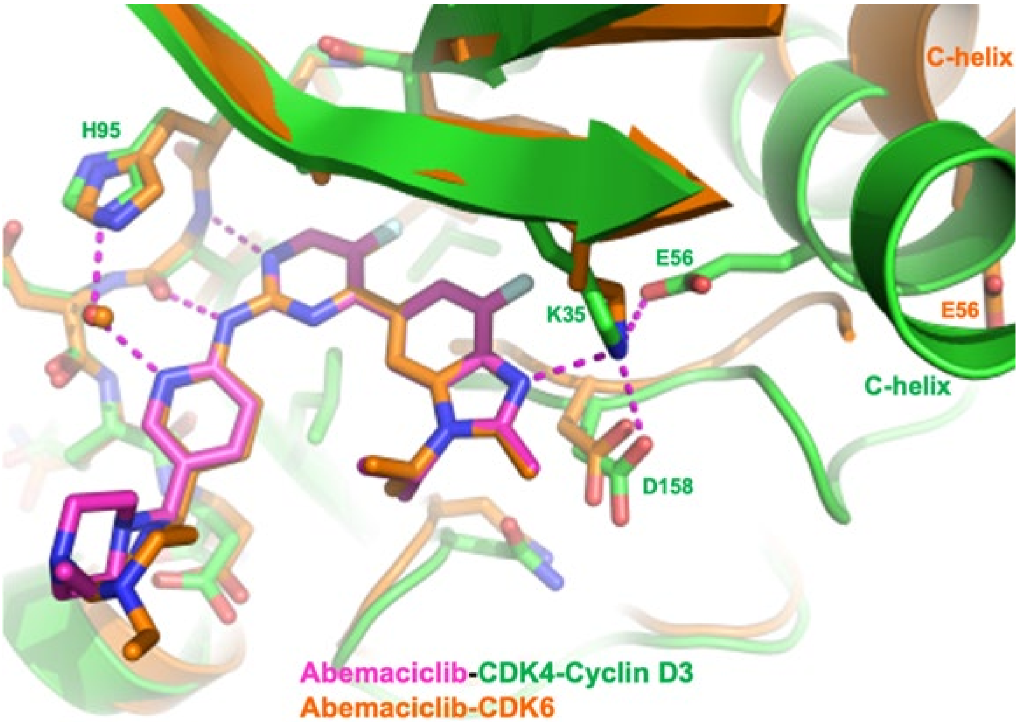

Supplementary Figure 4. Interactions of abemaciclib with the ATP site of CDK4-cyclin D3. The aminopyrimidine ring Hydrogen bonds with the backbone of residue V96. The pyridyl group binds through a water-mediated Hydrogen bond with H96. The free amine of the benzimidazole shares a Hydrogen bond with K35. The abemaciclib-CDK6 structure is shown superposed in orange (5L2S.pdb). Although the interactions are similar, the lack of Cyclin in the CDK6 structure results in a C-helix "out" conformation, and differences in the activation segment conformation.

Supplementary Figure 5

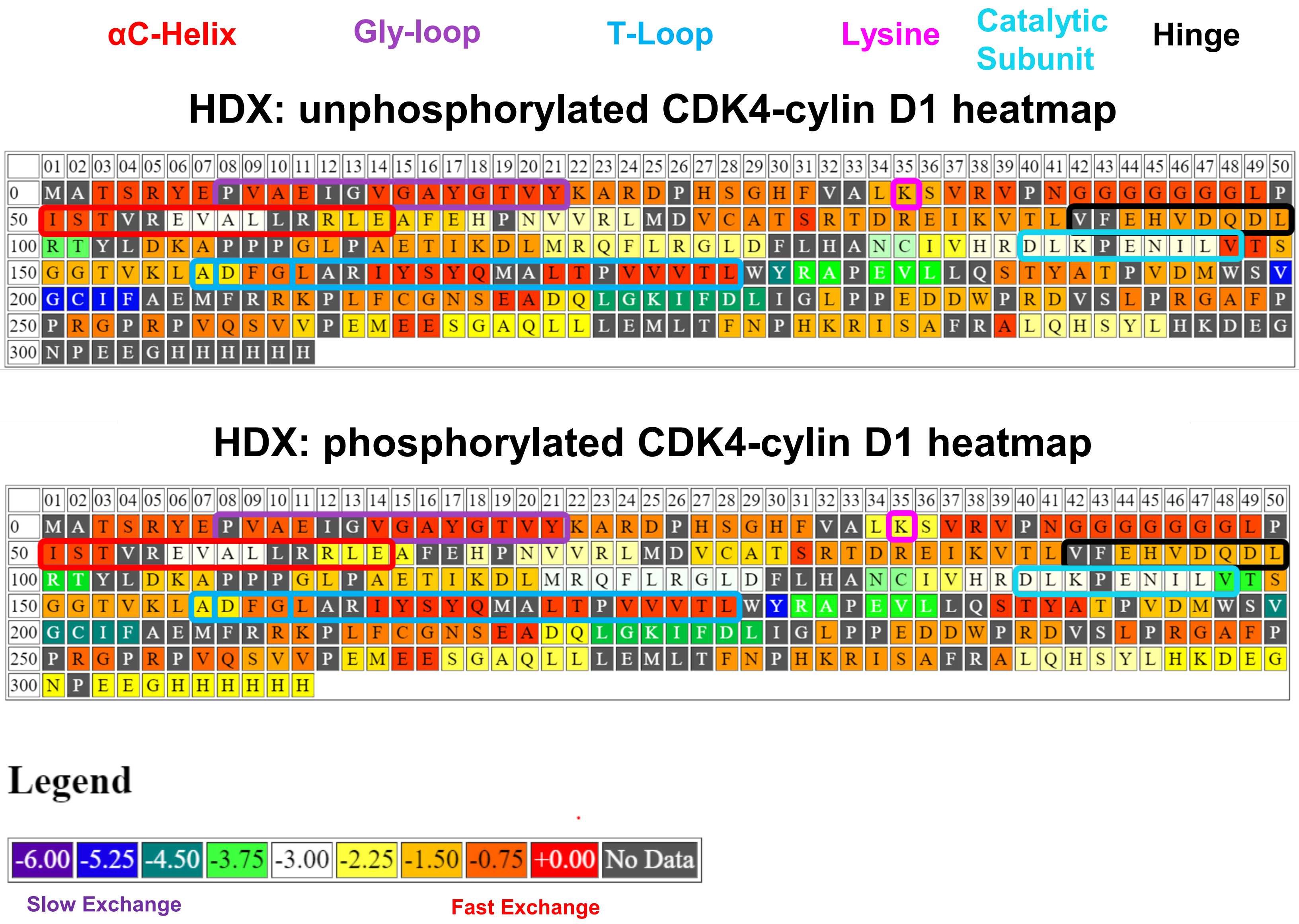

Supplementary Figure 5. HDX-MS heatmaps of unphosphorylated and phosphorylated CDK4-cyclin D in the absence of abemaciclib. CDK4 domains are highlighted and the DFG motif is shown within the T-loop.

Supplementary Figure 6

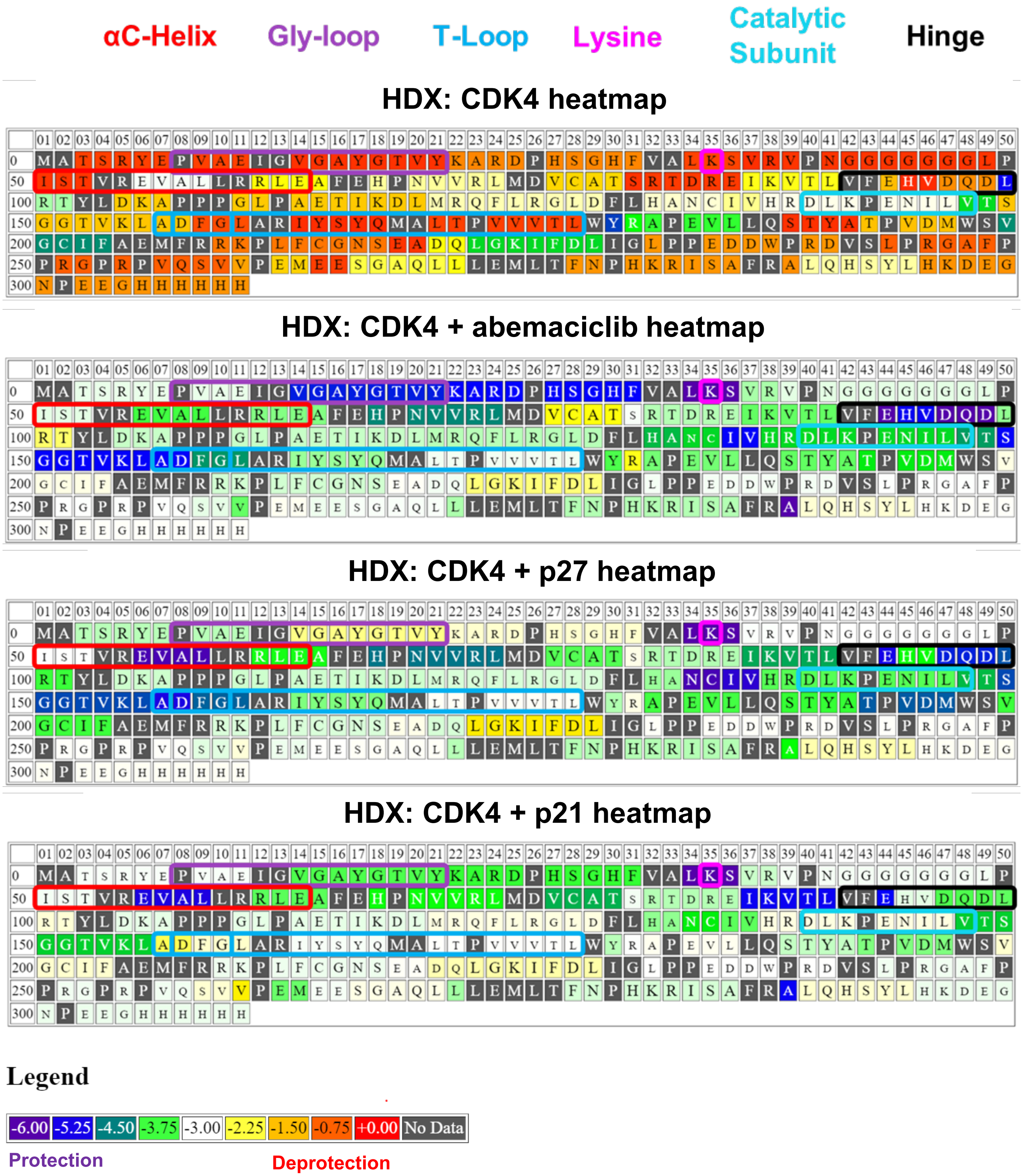

Supplementary Figure 6. HDX heatmaps of CDK4 alone and differential maps of CDK4-cyclin D single ligand experiments with either abemaciclib, p27 or p21, compared to CDK4 alone. CDK4 domains are highlighted and the DFG motif is highlighted within the T-loop.

Supplementary Figure 7

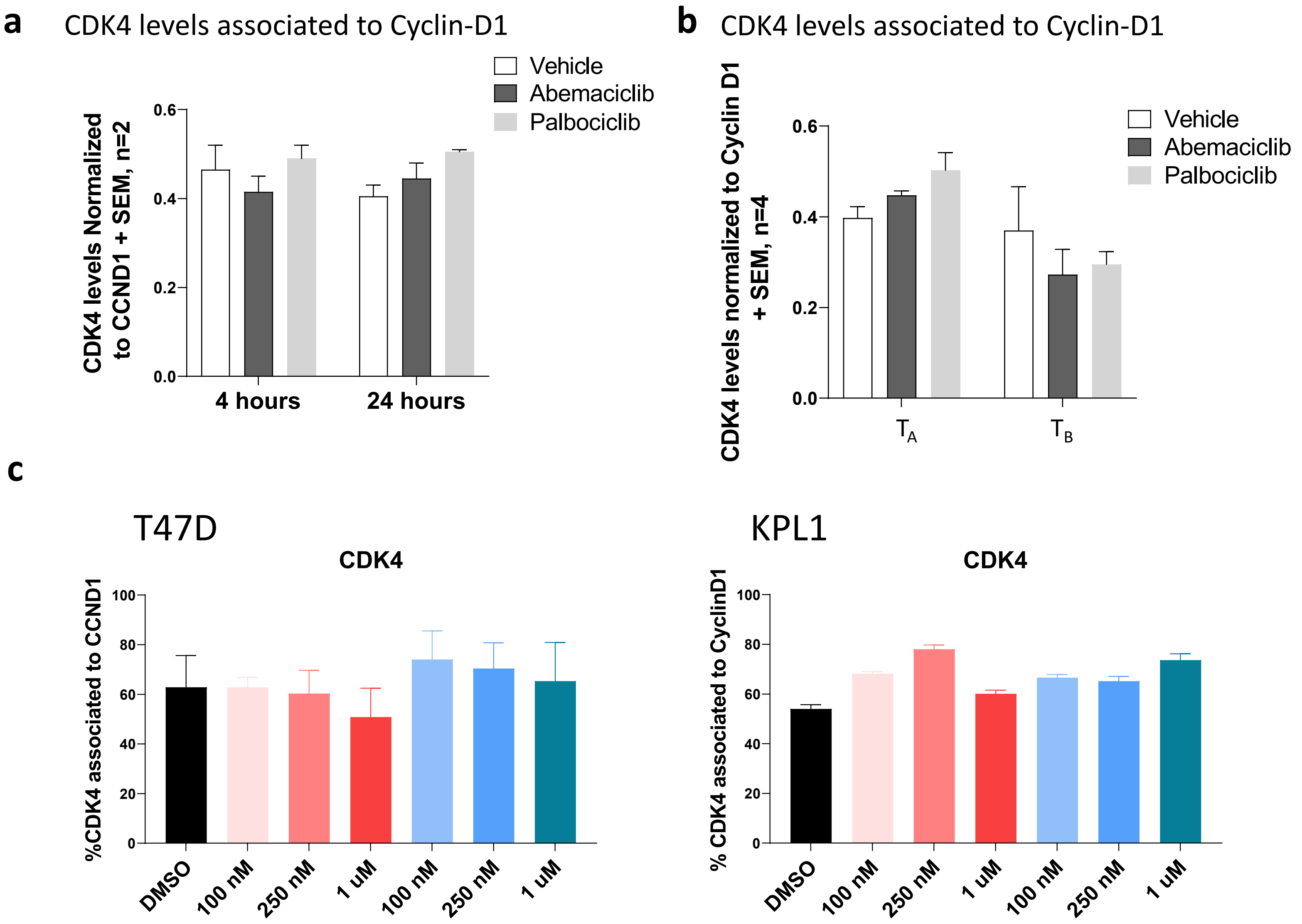

Supplementary Figure 7: Quantification of CDK4 associated with cyclin D1 following immunoprecipitation was monitored by LC-MS/MS. This data was generated from experiments reported in: **a** = Figure 3A and 4A; **b** = Figure 3B and 4B; **c** = Figure 5D. Error bars represent ± standard error

Supplementary Figure 8

Focused on ATP pocket

pT172 CDK4-cyclin D1-p21 projection | **pT172 CDK4-cyclin D1-p27 projection**

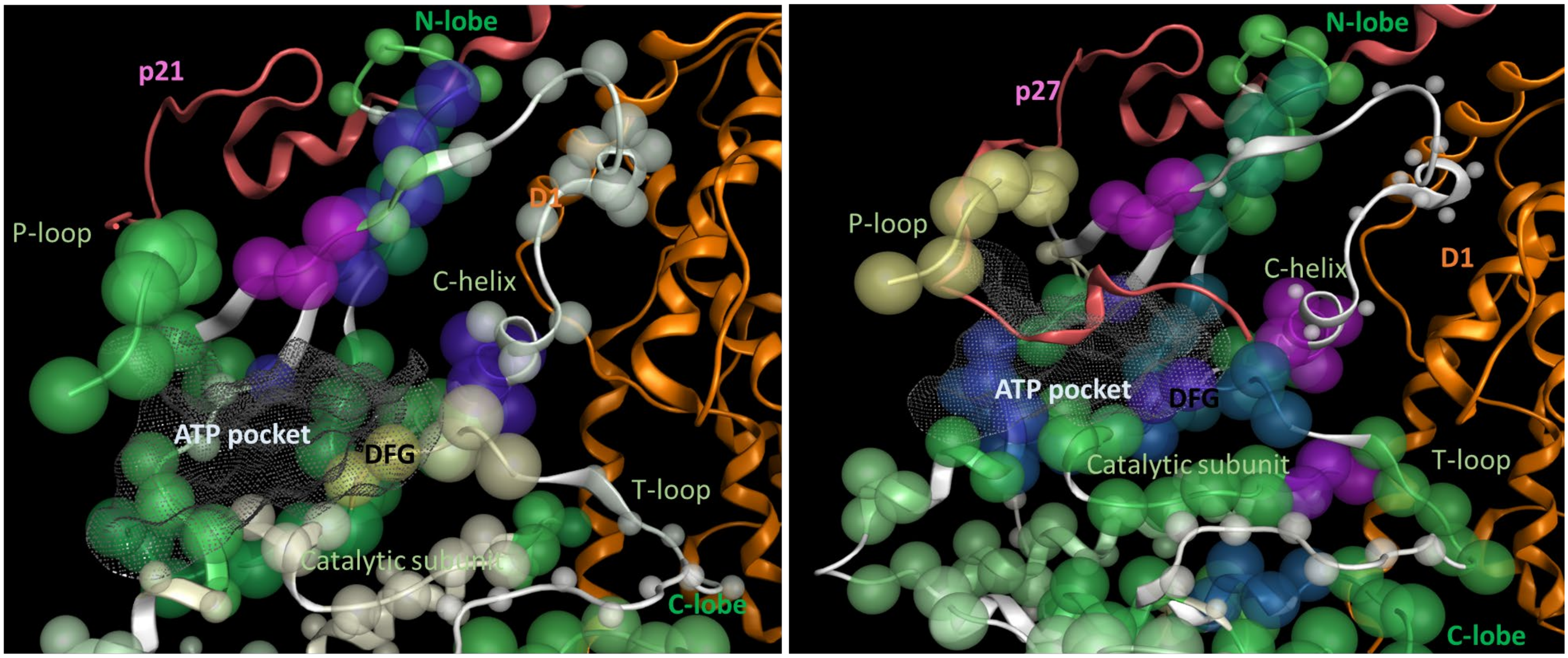

Supplementary Figure 8. Projection of pT172 CDK4-cyclin D1-p21/p27 focused on ATP pocket. CDK4 domains are highlighted and the DFG motif is shown within the T-loop. More blue hues indicate increased protection, less flexibility. More red hues indicate less protection, increased flexibility.

Supplementary Figure 9

Inhibitor	Target	Inhibition (%)	Bottom	Top	IC_{50} (pM)	Hill slope	S/B	Z-value
p21	CDK4/cycD1	98.5	5.9	100.3	121.56	1.92	8.8	0.73
p27	CDK4/cycD1	99.9	7.7	100.9	32.78	2.49	8.8	0.73

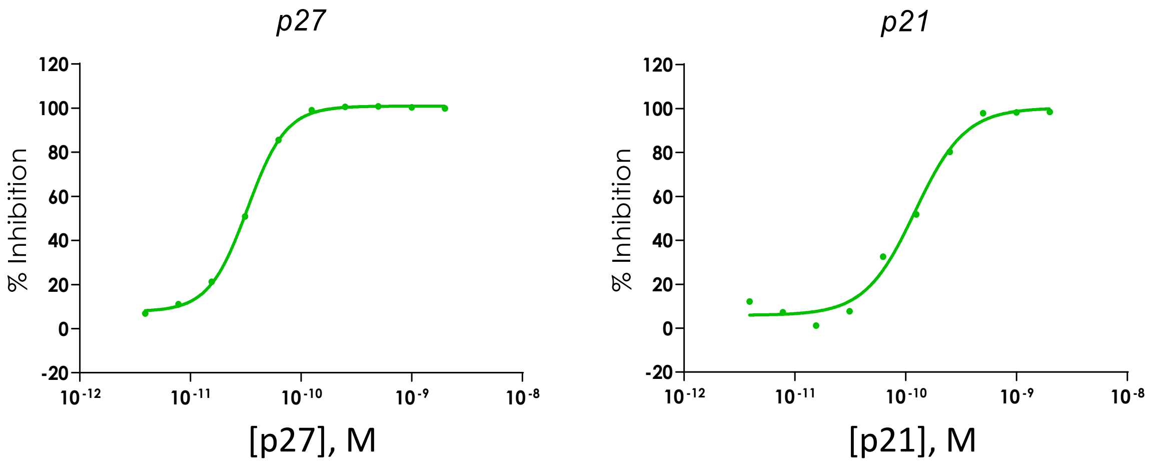

Supplementary Figure 9: p21 and p27 inhibits the activity of CDK4 activity *in vitro*. Biochemical analysis of the effect of p21 and p27 peptides in the kinase activity of CDK4/cyclinD1 recombinant protein.

Supplementary Figure 10a

p21 followed by abemaciclib projected in our internal x-ray

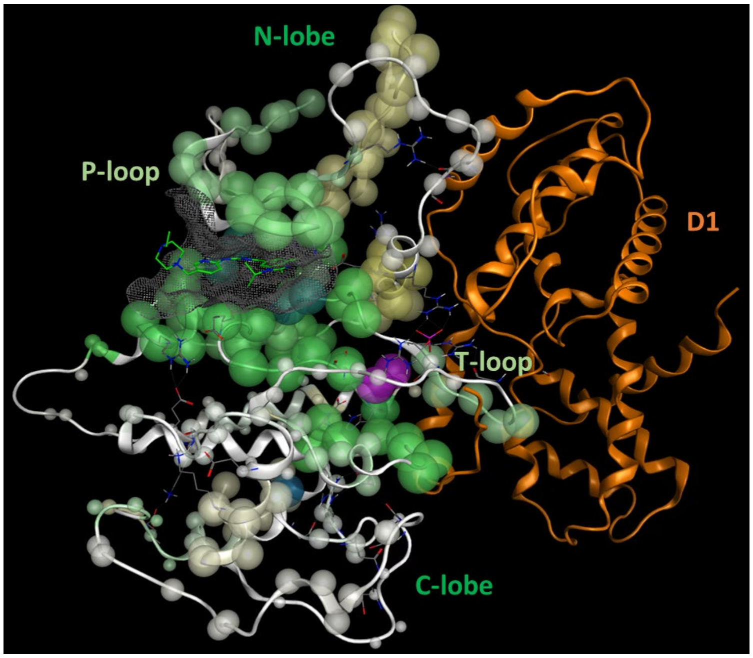

p27 followed by abemaciclib projected in our internal X-ray

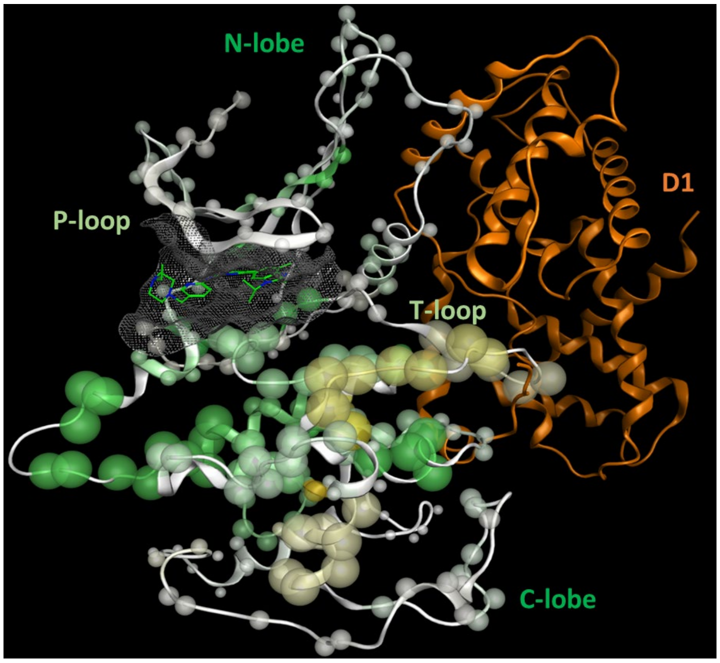

These two projections were built in our internal x-ray supposing that abemaciclib would displace p21 and p27 and for helping in the comparison. More blue hues indicate increased protection, less flexibility. More red hues indicate less protection, increased flexibility.

Abemaciclib followed by p21 projected in the model

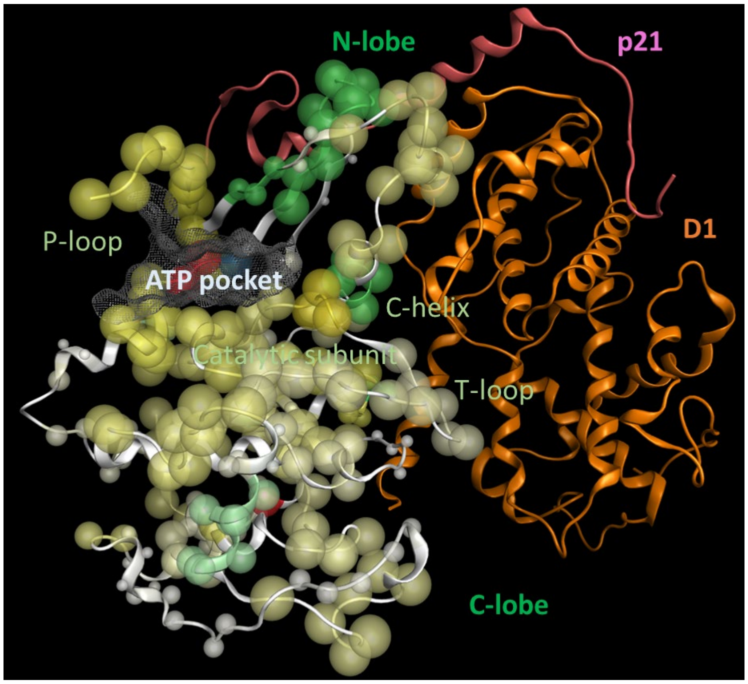

Abemaciclib followed by p27 projected in the model

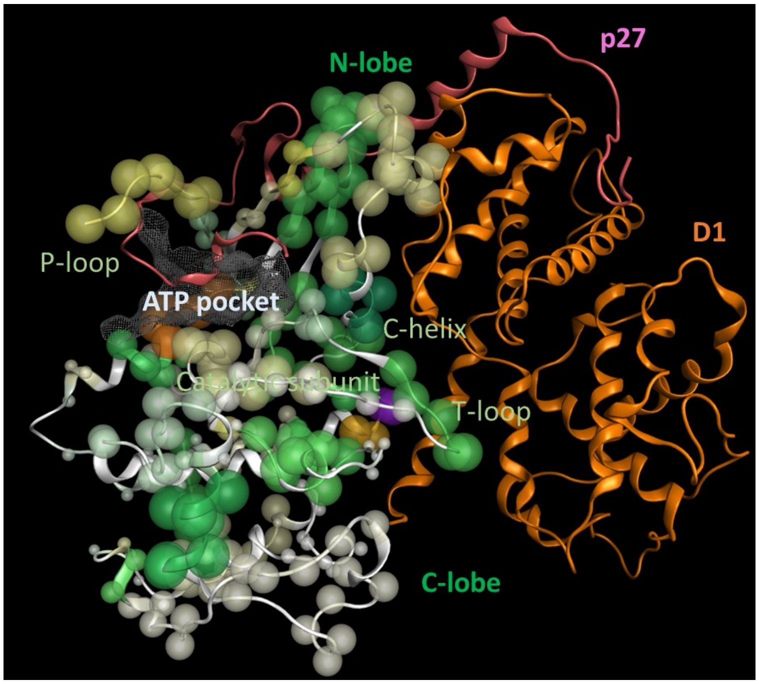

This two projections were built in models built CDK4-D1-p21/p27 supposing that p21 or p27 displaced abemaciclib and for helping in the comparison. More blue hues indicate increased protection, less flexibility. More red hues indicate less protection, increased flexibility.

Supplementary Figure 10b

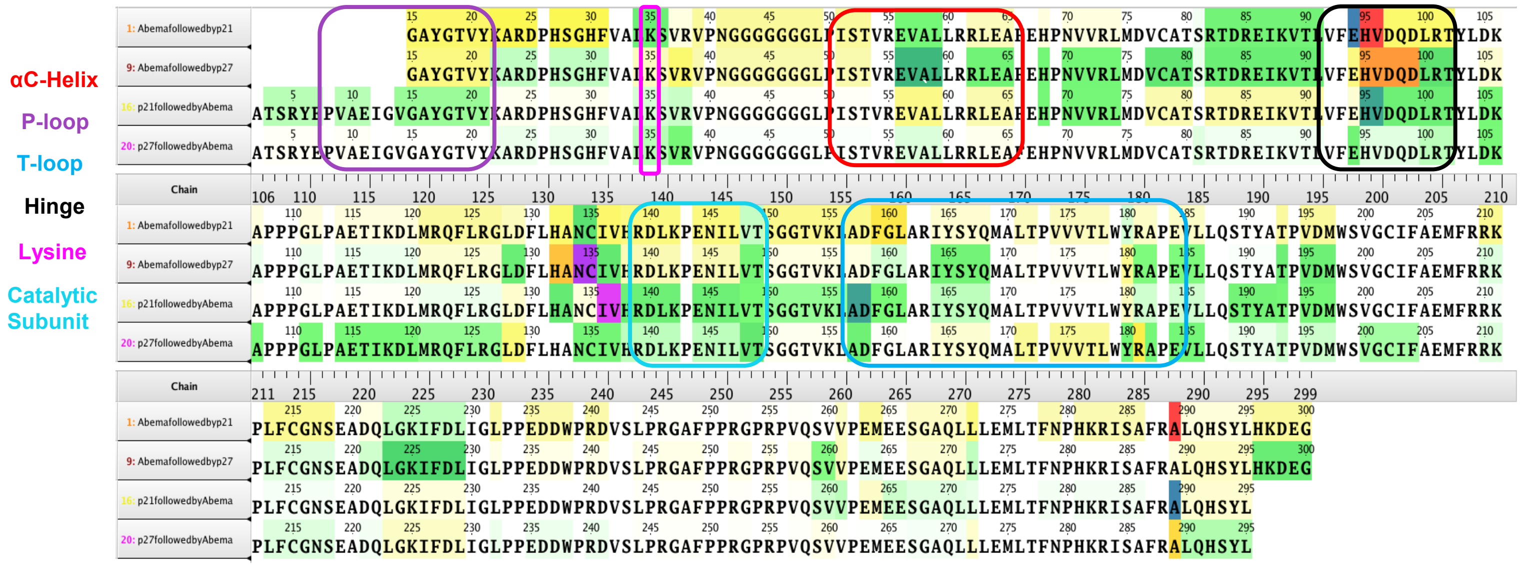

Supplementary Figure 10. (a) Projection and **(b)** sequence and domain alignment HDX-MS differential maps of p21/p27 and abemaciclib sequential binding. CDK4 domains are highlighted and the DFG motif is shown within the T-loop. Bluer hues indicate increased protection, less flexibility. More red hues indicate decreased protection, greater flexibility. font color is chosen only for optimum contrast with the background.

Supplementary Figure 11

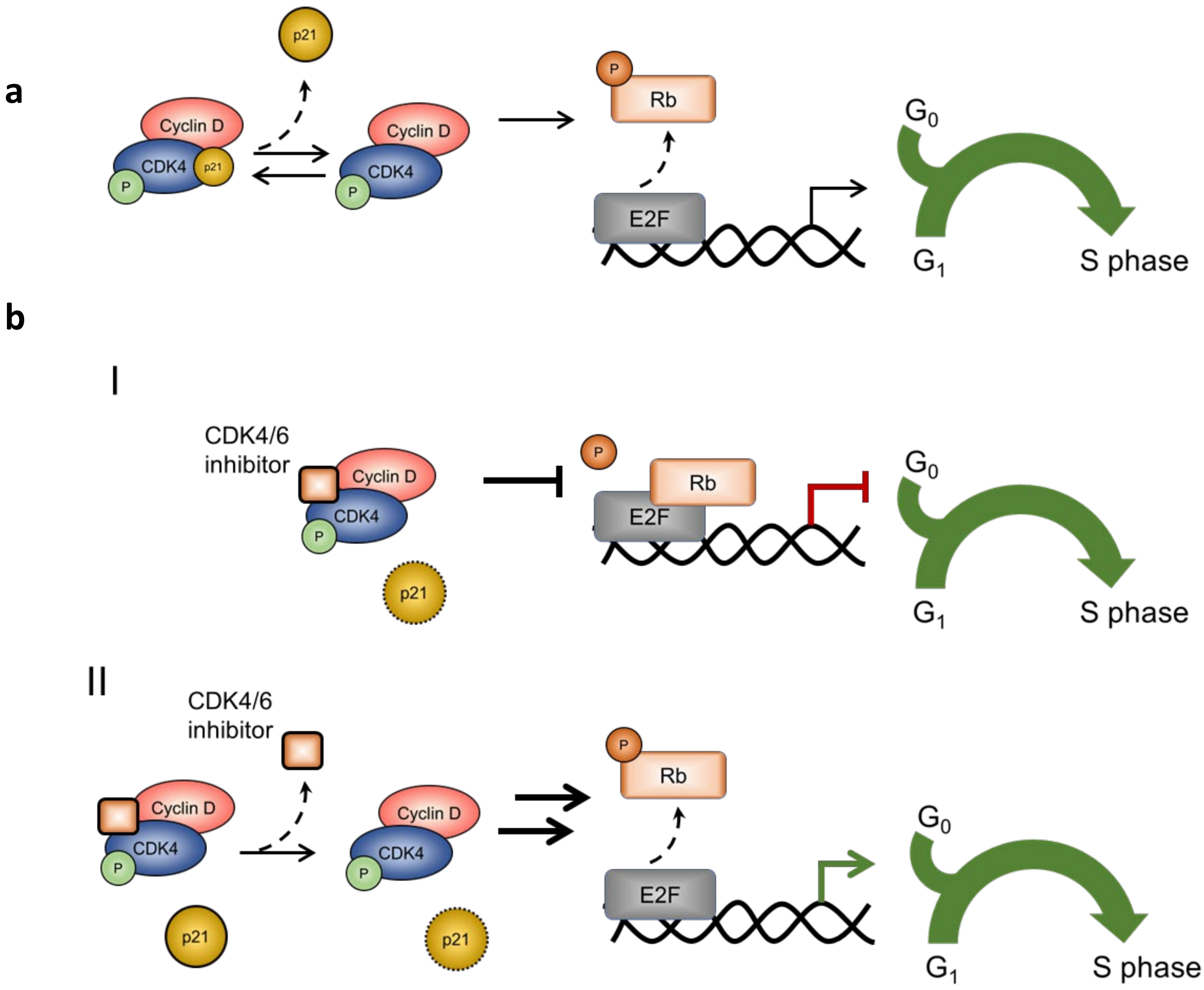

Supplementary Figure 11: Proposed model differentiating the effects of continuous versus intermittent treatment with CDK4/6 inhibitors. (a) In the absence of CDK4/6 inhibitors active CDK4 pT172-cyclin D complex phosphorylates and inactivates Rb, allowing for the release of E2F family of transcriptional factors, cell cycle progression and tumorigenesis. **(b)** We suggest a novel model of CDK4/6 inhibitor-mediated stabilization of **(I)** primed (active, but not operative) CDK4 pT172-cyclin D complex, demonstrating **(II)** rapid reversal and rebound activation of the CDK4 signaling pathway following abemaciclib removal highlighting the benefits of (B. I) continuous versus (B. II) intermittent CDK4/6 inhibitor dosing. In B. II, the dashed line around p21 indicates that p21 may or may not be bound to complex following abemaciclib removal.

Supplementary Figure 12

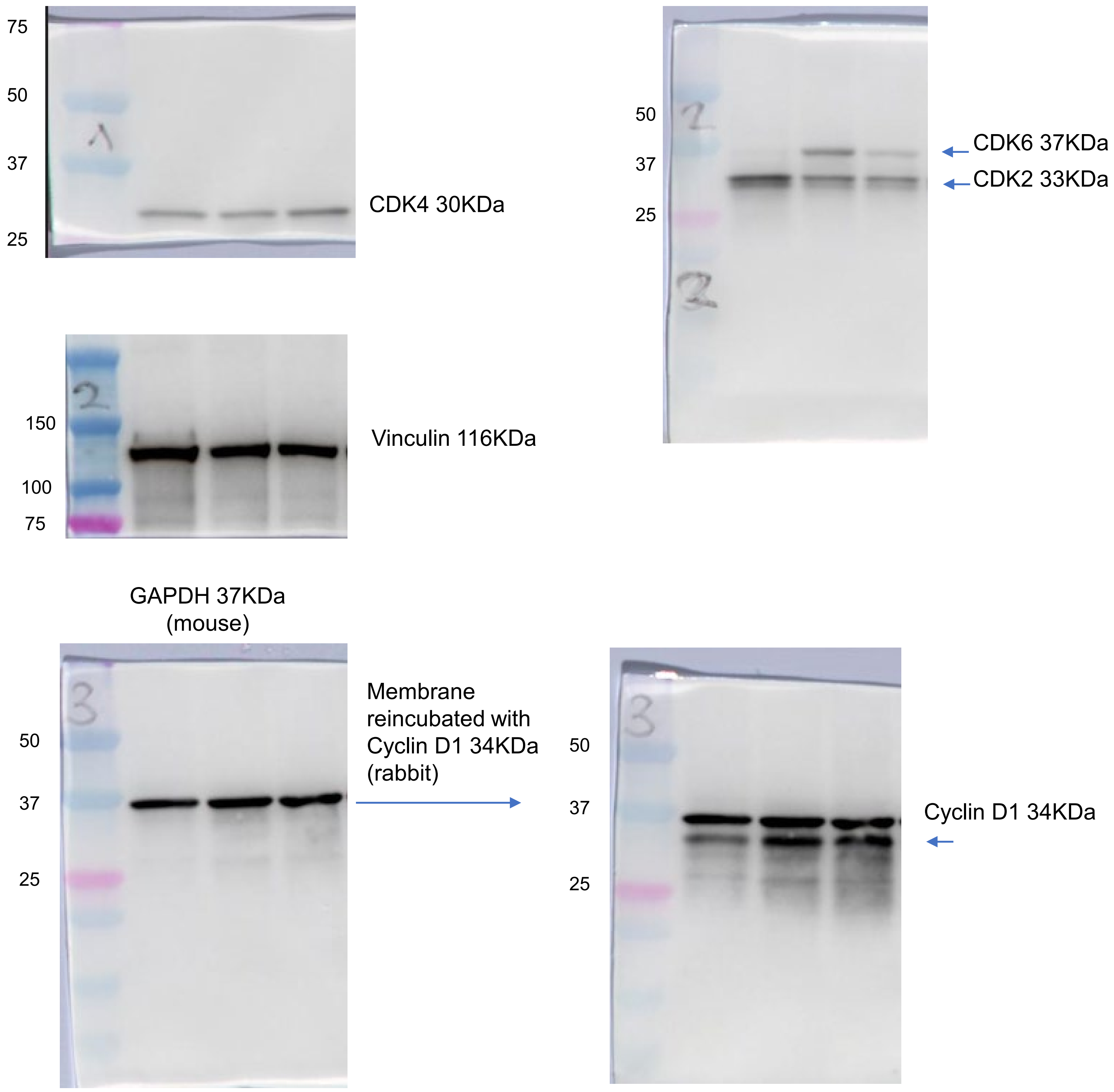

Supplementary Figure 12: Unmodified gel images related to Figure 5B.
